# Hybrid experimental and machine learning approach for optimizing abrasive wear of microcrystalline cellulose modified hemp/bamboo fiber composites

**DOI:** 10.1038/s41598-025-26396-0

**Published:** 2025-11-26

**Authors:** S. J. Davis Hans, M. Muthukumaran, K. Kumaresan, V. G. Pradeep Kumar, Dayanand M. Goudar, Subraya Krishna Bhat

**Affiliations:** 1 Department of Mechanical Engineering, Jansons Institute of Technology, Coimbatore, India; 2 Department of Mechanical Engineering, Park College of Technology, Coimbatore, India; 3 Department of Mechanical Engineering, JSS Science and Technology University, Mysuru, 570006 India; 4 Department of Mechanical Engineering, Tontadarya College of Engineering, Gadag, 582101 India; 5 Department of Mechanical and Industrial Engineering, Manipal Institute of Technology, Manipal Academy of Higher Education, Manipal, 576104 Karnataka India

**Keywords:** Hemp/bamboo-epoxy composites, Microcrystalline cellulose, Abrasive wear prediction, Machine learning, Engineering, Materials science

## Abstract

In this work, hemp/bamboo hybrid fabric–epoxy composites reinforced with 0–9 wt% microcrystalline cellulose (µCC) is examined for their abrasive wear behavior. Compression molding was used to create composites with 0, 3, 6, and 9 wt% µCC. In accordance with ASTM G65 guidelines, wear tests were conducted under controlled dry sand abrasion. Using a Taguchi L_16_ design, the effects of applied load (5–20 N), abrading distance (250–1000 m), and µCC content on wear loss were assessed. To predict abrasive wear and examine the role of µCC filler, several machine learning models were used, including Linear Regression, K-Nearest Neighbors, Artificial Neural Networks, Random Forest, Gradient Boosting, and eXtreme Gradient Boosting. By increasing the hardness and load-bearing capacity of the composite, µCC mechanistically increases wear resistance and lessens material removal during abrasion. According to ANOVA results, wear loss was most affected by abrading distance (44.08%), load (34.21%), and µCC content (18.01%). The Random Forest model had the lowest error (RMSE = 0.045) and the highest predictive accuracy (R^2^ = 0.942). Abrading distance is the main factor influencing wear resistance, followed by load and µCC content, according to feature importance analysis. Accurately forecasting abrasive wear and creating high-performance, sustainable hybrid composites can be accomplished by combining machine learning and experimental data.

## Introduction

Biocomposites are being widely studied due to the growing demand for sustainable materials. These materials replace synthetic reinforcements by combining renewable natural fibers with polymer matrices^1^. Natural fiber-reinforced polymer composites made using fibers such as hemp, bamboo, jute, and other cellulosic fibers offer several advantages, including low density, biodegradability, and reduced environmental impact^1,2^. Among them, hybrid fiber systems consisting of hemp and bamboo have shown great potential^3,4^. When these fibers are modified with fillers such as microcrystalline cellulose (µCC), their tribological and mechanical properties can be further improved^5,6^.

Natural fiber-reinforced composites (NFRCs) have gained significant attention as sustainable alternatives to conventional materials. Several reviews have discussed the recent advancements and persistent challenges in this field. Mohammadi et al.^7^ reported that the incorporation of micro- and nano-fillers can considerably enhance the strength, durability, and overall performance of NFRCs. However, they also identified challenges such as nanofiller scaling, poor interfacial adhesion, and inadequate dispersion that hinder large-scale industrial adoption. Olanrewaju et al.^8^ emphasized the potential of NFRCs as eco-friendly substitutes for synthetic fiber composites. Their review highlighted approaches to improve mechanical and thermal properties by addressing issues such as hydrophilicity, weak fiber–matrix bonding, and moisture sensitivity. Similarly, Ayana et al.^9^ critically reviewed wood polymer composites (WPCs) as examples of sustainable construction materials. They demonstrated that the use of recycled plastics, fiber–matrix modifications, and optimized formulations can enhance the environmental performance of WPCs. Collectively, these studies illustrate a growing research effort aimed at overcoming the limitations of natural fiber composites while maximizing their potential as high-performance, sustainable materials.

Direct cutting or ploughing action results from hard particles or asperities that are firmly adhered to one surface and slide against another in two-body abrasive wear (2-BAW)^10^. Applications such as metal machining and gear contact frequently use this mechanism. Three-body abrasive wear (3-BAW), on the other hand, results in rolling, sliding, and indentation effects because loose abrasive particles, like sand, are trapped between two sliding surfaces. This wear mechanism commonly occurs in applications involving soil contact, construction machinery, power plant components, and agricultural implements^11,12^. Owing to the random motion of free abrasive particles, three-body abrasive wear (3-BAW) often causes more complex and severe material removal.

In fiber-reinforced polymer composites, wear behavior depends on fiber type, orientation, interfacial bonding, and the presence of fillers. Strong bonding between fiber and matrix helps resist material removal, while weak bonding can cause fiber pull-out, matrix cracking, and surface damage^13,14^. Adding hard or lubricating fillers, such as silica, alumina, graphite, or µCC, improves wear resistance by enhancing load transfer and reducing friction^15,16^. Carbon fiber-reinforced ultra-high molecular weight polyethylene (UHMWPE) composites have improved mechanical and tribological properties, as demonstrated by Ventura et al.^17^. Combining different natural fibers, such as hemp and bamboo, further improves wear resistance due to the synergistic interaction of fibers with different mechanical properties. Saha et al.^18^ studied sustainable brake friction composites using bio-based fibers, resins, and natural additives instead of hazardous metals. This approach improves performance while reducing micro-plastic and wear debris generation, addressing environmental concerns. Together, the two studies show a distinct tendency toward the integration of sustainable elements without compromising material performance. Thus, filler content and fiber–matrix adhesion is therefore crucial to developing natural fiber composites with superior tribological performance. Overall, optimizing filler content and fiber–matrix adhesion plays a key role in developing natural fiber composites with superior tribological performance.

Little research has been done on 3-BAW, especially in bio-based hybrid systems, despite the growing interest in natural fiber-reinforced polymer composites. The wear of hemp/bamboo hybrid fabric reinforced epoxy composites modified with µCC under dry sand abrasive conditions is examined in this study to close that gap. By decreasing experimental dependency, cutting costs, and speeding up material development for tribological applications in the construction, automotive, and defense industries, the incorporation of machine learning (ML) for predictive modeling further advances the field.

In applications where sand or hard particles contact is frequent, the durability of polymer composites is greatly impacted by 3-BAW behavior. Although abrasive wear in natural fiber polymer composites under dry sand conditions has been studied in several studies, the majority of them rely on intensive experimental testing^19–25^. Numerous experimental investigations have examined 3-BAW in composites reinforced with natural and synthetic fibers, highlighting the influence of matrix interactions, filler content, and fiber type. While Kumaresan et al.^19^ and Manoharan et al.^20^ illustrated the wear resistance of synthetic reinforcements and fillers in carbon/epoxy and brake pad composites, Mishra and Biswas^21^ illustrated the significance of fiber orientation in jute/epoxy systems. As demonstrated by Rajashekaraiah et al.‘s work^22^, thermoplastic systems also demonstrated encouraging wear resistance. More recently, Darshan et al.^23^ used statistical modeling and nanofillers to optimize wear in silk/basalt hybrid composites. The potential of hybrid green composites to provide balanced mechanical and tribological performance is highlighted by complementary results from Suresha et al.^24^ and Kumar et al.^25^. These studies highlight the importance of hybrid hemp–bamboo fibers and µCC fillers in improving 3-BAW resistance under dry sand conditions. By increasing filler–matrix adhesion and reducing material loss under abrasive loads, embedding fillers like micro or nano-cellulose improves hardness and wear resistance^26^. However, there is currently no research on predictive models specifically designed for hemp-bamboo/µCC reinforced systems.

Research on polymer composites has quickly embraced ML techniques, which allow for the predictive modeling of mechanical, thermal, and tribological properties depending on composition and loading circumstances^2,27^. Particularly in tribology, ML methods like Gradient boosted regression (GBR), Random Forest regression (RFR), and artificial neural networks (ANN) have successfully predicted the friction and wear rate of polymer-based composites reinforced with particulates (fly ash), obtaining high R^2^ values (> 0.90)^27,28^. For example, RFR and gradient-boosted models reduced the experimental burden by accurately predicting wear behavior in epoxy coatings and polymer composites^29–31^. Ibrahim et al.^32^ demonstrated the usefulness of hybrid ML models for predicting the wear behavior of PTFE matrix composites. They used models such as Taguchi–GRA integrated with SVR–particle swarm optimization (PSO) and support vector regression combined with Harris Hawks’ optimization (SVR–HHO). Among these, SVR–HHO showed the best prediction accuracy and fit, highlighting its potential for optimizing tribological materials. Prabhu et al.^33^ reported that adding TiO₂ filler significantly improved the abrasive wear resistance of flax fiber–reinforced epoxy composites, with filler content and grit size being key factors. The Taguchi model’s dependability in maximizing wear performance for sustainable natural fiber composites was confirmed by the investigation.

The dry sliding wear behavior of basalt fabric-reinforced epoxy composites, both with and without nano-Al₂O₃, was investigated^34^. Wear resistance increased by about 16.9% with the inclusion of nanoparticles, with load and hardness being the most important variables. Their findings demonstrated that compared to multiple regression analysis (MRA), the ANN model produced more accurate wear predictions. Due to better interfacial bonding and load transfer, Singh et al.^35^ reported that adding MWCNTs to reinforcing glass fiber-reinforced polymer composites greatly improved their mechanical and tribological properties. Its application in material design is supported by the ANN model’s precise prediction of the friction coefficient. Similarly, Jain et al.^36^ employed ensemble machine learning approaches to forecast MWCNT modified PMMA nanocomposites tribological performance. The GBM model showed promise in reducing experimental efforts in nanocomposite synthesis, outperforming random forest and additional trees with an R^2^ of 0.99.

The literature highlights the significance of sustainable biocomposites that reduce density, improve biodegradability, and minimize environmental impact by blending renewable natural fibers with polymer matrices. Particularly under abrasive wear conditions, hybrid architectures reinforced with fillers such as µCC show notable improvements in mechanical and tribological properties. Improving wear resistance and durability in bio-composites requires optimizing fiber–matrix bonding, filler content, and hybrid designs, often using techniques like Taguchi optimization, as shown in studies on 2-BAW. Compared to 2-BAW, 3-BAW, which more accurately mimics real-world abrasive conditions with loose particles, is still not well understood in bio-based hybrid composites. Current studies demonstrate that by enhancing fiber-matrix bonding and surface hardness, hybrid fiber architectures with micro/nano-fillers improve abrasion resistance. However, the literature on predictive models for hemp-bamboo/µCC modified epoxy composites under 3-BAW is noticeably lacking, especially when it comes to applying ML techniques for precise wear prediction.

The research gap is the lack of ML-driven predictive frameworks for 3-BAW unique to epoxy composites reinforced with hemp-bamboo fibers modified with µCC. Most existing studies rely on traditional experimental methods, which are time-consuming and require many resources, instead of using data-driven modeling for faster material optimization. Machine learning (ML) techniques have shown promise in predicting the tribological and mechanical performance of various polymer composites. However, their application to hybrid natural fiber composites under 3-BAW conditions has not yet been explored.

The effective prediction of intricate wear responses made possible by the incorporation of ML into the study of natural fiber composites optimizes material design while reducing the need for expensive testing^2,27–36^. The goal of this study is to develop a thorough, ML-based predictive model for 3-BAW behavior in epoxy composites with hybrid hemp-bamboo fibers and different amounts of µCC filler. These composites will be tested under ASTM G65 standard dry sand abrasion conditions. This study predicts wear loss based on key factors such as filler loading, applied load, and abrading distance. It compares the performance of several supervised machine learning algorithms, including linear regression, K-nearest neighbors, artificial neural networks, random forests, gradient boosting, and extreme gradient boosting.

The objectives of this research were to develop and evaluate hemp-bamboo hybrid epoxy composites with different loadings of µCC to produce useful datasets for training ML models.

This study combines machine learning with Taguchi design of experiments (DoE) to create a reliable tool for predicting abrasive wear in hemp/bamboo hybrid fabric–epoxy composites reinforced with µCC filler. The method not only optimizes experimental parameters but also compares results with the Ratner-Lancaster model and relates them to hardness data, providing a complete way to assess wear performance. The overall goal is to develop an ML-based approach for designing tribological materials, reducing experimental effort, and advancing sustainable composites for high-wear engineering applications. This research has potential applications in agricultural tools exposed to soil wear, lightweight and abrasion-resistant defense equipment, automotive parts, wear-resistant building materials, and eco-friendly alternatives to synthetic fiber composites that reduce carbon emissions and support the circular economy.

## Materials and methods

### Materials

#### Epoxy resin and hardener

The epoxy resin is Araldite MY740, and the hardener is Anhydride HY918. Both are products from Huntsman Limited USA. A common method for producing a thermosetting polymer matrix is this combination. To produce a stiff, long-lasting material, the resin and hardener are combined and go through a chemical process known as polymerization. The purpose of this matrix is to hold the fiber and fillers together.

#### Hybrid fabrics

Hemp and bamboo fibers are combined to form the hybrid fabric (H/B F; 140 GSM), a composite reinforcement material. It was obtained from India’s Pahartah Fashion LLP. Here 140 GSM refers to the fabric’s areal density, or mass per unit area, which is 140 g per square meter. A focus on producing a more sustainable or bio-based composite is suggested using hemp and bamboo fibers in plain weave woven mat form.

#### Microcrystalline cellulose

The highly refined wood pulp known as microcrystalline cellulose (µCC) is utilized in this application as a reinforcing filler It is characterized as a very fine powder, having an average particle size of under 20 μm. It can be identified as a particular chemical compound by its CAS number, 232-674-9. µCC which is supplied by MeRCK, USA, can be added to epoxy resin to change its characteristics. It can improve the composite’s dimensional stability, stiffness, mechanical strength and resistant to wear while potentially lowering the material’s overall cost.

### Composites fabrication

Existing research indicates that µCC contents above 10 wt% frequently result in agglomeration, poor fiber wetting, and reduced mechanical integrity because they reduce the effectiveness of stress transfer between the matrix and reinforcement phases^37,38^. Additionally, a small range promotes uniform interfacial bonding and ideal dispersion, which strengthens the hybrid system’s tribo-mechanical stability.

According to established bio-composite formulations, a total fiber loading of 40 wt% (bamboo/hemp) was used in this study to ensure optimal wetting and strong interfacial adhesion within the textile-grade hybrid composite system^39,40^. To systematically clarify its impact on composite properties while keeping dispersion and viscosity within acceptable bounds during compression molding, µCC was added at concentrations ranging from 0 to 9 wt%^41^. This concentration range allows comparison with previous studies that found that adding µCC up to 5–10 wt% to epoxy and natural fiber composites improved stiffness, thermal stability, and wear resistance^42,43^. The ASTM G65 method, which offers a consistent and repeatable evaluation of dry sand abrasion under regulated load and abrasive feed conditions, was used to assess the wear performance. This ensures a consistent and trustworthy characterization of the tribological behavior of the composites.

**Fig. 1:**
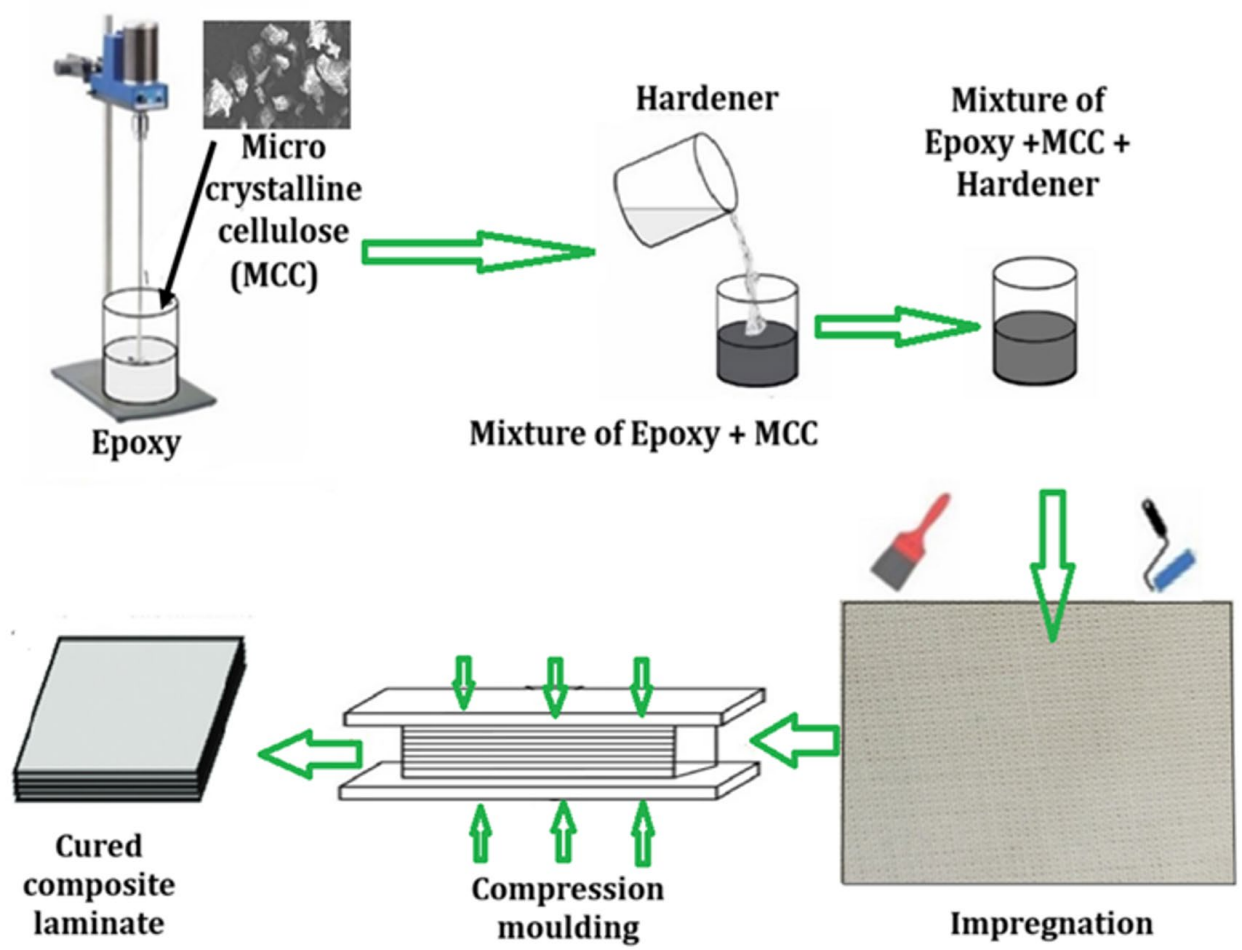
Fabrication process of H/B F-Ep composites with µCC particulates.

The composite was made using compression molding. In this process, a set amount of reinforcement (12 H/B fibers and 60 wt% epoxy) is placed in the mold, and the epoxy–hardener mixture is poured into the heated mold cavity. Once the mold is closed, pressure is applied, compelling the material to conform fully to the mold cavity and thereby taking on its final shape (Fig. 1).

Bamboo fibers are aligned in a 90^o^ orientation, while hemp fibers are aligned in a 0^o^ orientation, creating a hybrid fabric that serves as reinforcement. The cross-ply layup, as it is commonly called, is a strategic arrangement intended to enhance the mechanical characteristics of the composite in particular directions. Bamboo fibers oriented 90^o^ would add strength perpendicularly, whereas hemp fibers oriented 0^o^ would mainly contribute strength along that axis. This design makes it conceivable to modify the properties of the composites to meet load-bearing needs.

A few crucial steps were involved in the matrix preparation to ensure the quality of the finished composite. (i) Filler Dispersion: Microcrystalline cellulose (µCC) particles were first combined with epoxy resin. To ensure that the µCC particles were evenly distributed throughout the epoxy resin, a mechanical stirrer running at 3500 rpm was employed. To achieve consistent mechanical characteristics in the final cured composite, it is authoritative that the filler particles be appropriately dispersed to prevent clumping and create a homogenous mixture. (ii) Starting Polymerization: The hardener was added after the epoxy-µCC mixture was uniform. The crosslinking reaction, also referred to as polymerization, that turns the liquid resin system into a solid, rigid polymer matrix is started by the hardener, which is the curing agent. (iii) Final Mixing: The mixture was stirred once more after the hardener was added. To make sure that the hardener is dispersed uniformly throughout the epoxy-µCC system, this last mixing step is essential. For the composite to cure evenly and acquire its desired characteristics, all the ingredients i.e. epoxy, µCC, and hardener must be distributed evenly. After placing the mold with the impregnated layers in a hot press, compression molding was done for 20 min at 70 °C and 40 bars of pressure. This step enabled the matrix to flow and conform around the fibers as the curing process began. After initial molding, the composite underwent a two-stage post-curing process. The samples were initially kept at room temperature (23 ± 1 °C) for 24 h to allow the Ep to cure gradually. Thermal curing was then done for four hours at 80 °C to complete the polymerization reaction and enhance the mechanical properties of the composite.

Following curing, the result was a composite laminate that was packed with uniformly spaced µCC particles inside the epoxy matrix and reinforced with mats of hybrid hemp and bamboo fibers. This process offered enhanced interfacial adhesion, uniform filler dispersion, and ideal curing conditions for improved structural performance of the composite. Table 1 provides the wt% of the all the constituents of the H/B F-Ep composites and their µCC modified composites that were produced and examined in this investigation.

**Table 1:** H/B F-Ep composite with µCC filler selected for the present work.

Composites designation	Epoxy (wt%)	Textile fabric (wt%)	µCC (wt%)	Description
H0	60	40	-	No µCC, baseline textile hybrid composite
H1	57	40	3	Textile hybrid fabric + 3 wt% µCC
H2	54	40	6	Textile hybrid fabric + 6 wt% µCC
H3	51	40	9	Textile hybrid fabric + 9 wt% µCC

### Testing methods

#### Microscopy

The worn surfaces of the H/B F-reinforced epoxy (H/B F–Ep) composites were examined using field emission scanning electron microscope (FEG-SEM, LEO Supra 35, Zeiss, Wetzlar, Germany) operated at 5 kV with a secondary electron detector. The unfilled H/B F–Ep composite (H0) and its hybrid version (H3) were captured at different magnification to ensure comparability. To ensure imaging stability and focus precision, all specimens were mounted on aluminum stubs using conductive carbon tape after being sputter-coated with a 20 nm thick coating of gold to improve conductivity and reduce electron charging. Fiber–matrix and filler–matrix interfaces, filler dispersion, fiber alignment, and voids were all assessed using SEM observations.

#### FTIR

The chemical composition and bonding properties of the unfilled H/B F-Ep composite (H0) and its hybrid counterparts (H1 to H3) were examined using Fourier Transform Infrared Spectroscopy (FTIR) analysis. Fourier-transform infrared (FTIR) characterization was performed using a Perkin Elmer Frontier 4100 instrument equipped with a KBr beamsplitter. Spectra were collected over the wavenumber range of 4000 to 600 cm^− 1^at a resolution of 16 cm^− 1^. The instrument scanned samples at a rate of 2 mm s^− 1^ to ensure accurate spectral acquisition. For each analysis, 10 to 15 mg of finely ground powder were used to produce the composite samples. To evaluate filler-matrix interactions and chemical bonding within the H0 composite system, the technique detects molecular vibrations through infrared light absorption at frequencies that correspond to vibrational bond energies of atoms in the composite matrix, hemp fibers, and µCC filler.

#### Hardness

The Shore D hardness tester (Model: HT-6510D, Korea) was utilized to assess the hardness of the hybrid epoxy-based composites in accordance with ASTM D2240-00 guidelines^44^. The indentation depth was measured using a hardened steel indenter that had a 1.4 mm diameter, a 30° conical point, and a 0.1 mm tip radius. A constant load of 45 N was applied for each test. Tests of hardness were performed on flat specimens of H/B F-Ep composites, measuring 25 × 25 × 2.5 mm^3^, both with and without µCC particulates.

#### Three-body abrasive wear test

**Fig. 2:**
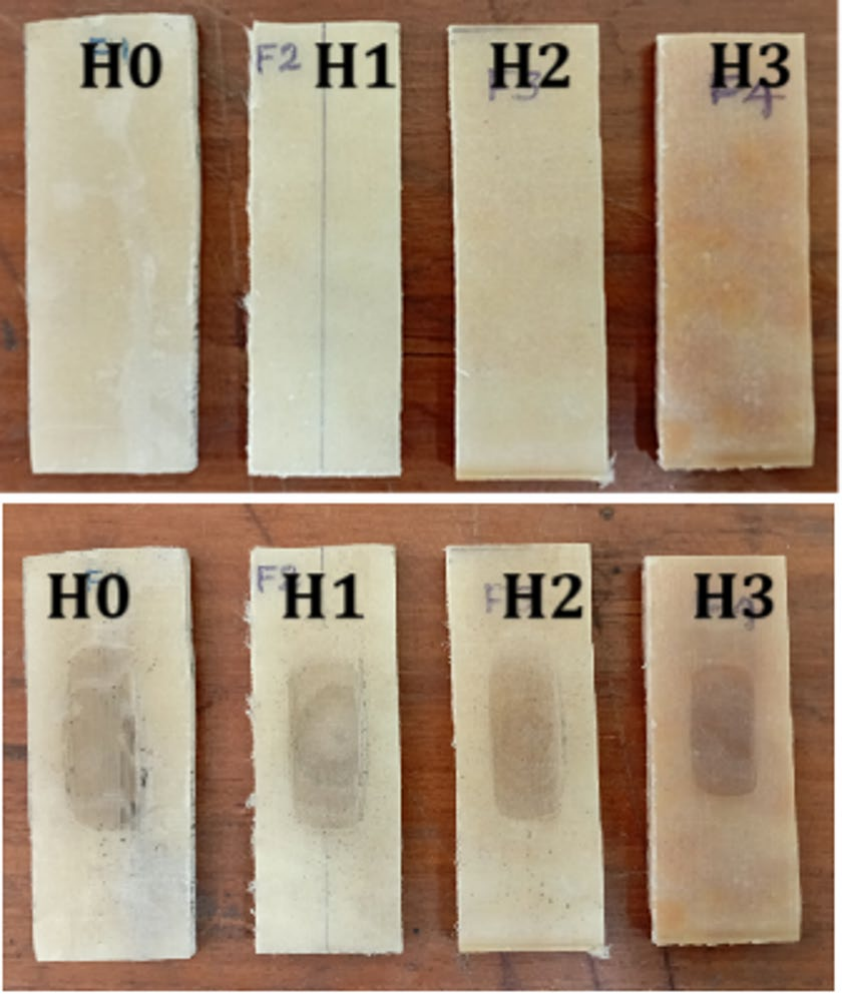
Specimens from 3-BAW test: (**a**) before and (**b**) after testing.

For the three-body abrasive wear (3-BAW) test, a dry sand-rubber wheel apparatus was used in compliance with ASTM G65 guidelines^45^. A controlled normal load was applied while the test specimen was fixed against a revolving rubber wheel. Throughout operation, a constant stream of standardized silica sand abrasive was fed between the wheel and the specimen. Controlled material removal was achieved by allowing the abrasive particles to freely roll and slide between the contacting surfaces. Test parameters like load, rubber wheel speed, abrasive feed rate, and test duration were fixed and predefined for each sample. To calculate the amount of weight loss brought on by wear, specimens were cleaned, dried, and weighed at the completion of each test. Several variables, including abrading distance, applied normal load, and µCC loading, were considered when calculating the wear loss (g). Figure 2a and b illustrate the selected specimens both before and after the 3-BAW testing.

### Machine learning approach

Using a random division technique, the dataset was separated into training and testing sets. To reduce sampling bias, the data was stratified to maintain the distribution of important variables like wear severity or other pertinent features. Specifically, 80% of the data was devoted to training and 20% to testing. To minimize overfitting and evaluate model robustness, cross-validation methods were used, such as k-fold cross-validation with k = 10. To average out variability and guarantee stability of the results, multiple training/testing splits were also carried out, with iterations usually occurring five times. By offering an objective assessment of predictive performance, these techniques aid in avoiding overfitting and improve the generalizability of the model.

#### Linear regression

This method aids in comprehending how input parameters affect the response of the output. A supervised machine learning technique called linear regression (LR) is used for predicting results based on input features^46^. For a given set of input variables $\:{x}_{1},{x}_{2},...,{x}_{p}$, the LR model can be expressed as (Eq. 1):

1 $$\:{y=\beta\:}_{0}+\sum\:_{j=1}^{p}{\beta\:}_{j}{x}_{j}+\epsilon$$

where $\:y$ represents the predicted response, $\:{\beta\:}_{0}$ is the intercept term, $\:{\beta\:}_{j}$ denotes the regression coefficient for the $\:{j}^{th}$ input variable, $\:p$ is the total number of input variables, and $\:\epsilon\:$ corresponds to the random error term. The coefficients $\:{\beta\:}_{j}$ are typically obtained by minimizing the residual sum of squares between the observed and predicted values^47^.

In this study, experimental parameters like filler loading, applied load, and abrading distance were used to predict three-body abrasive wear (3-BAW) using the Linear Regression model. These inputs and the observed wear loss or wear rate are directly correlated by the model. The obtained coefficients shed light on how each parameter affects the hybrid composite system’s wear behavior in relation to the others.

The LR model is limited in its ability to capture the nonlinear interactions present in tribological processes such as abrasion, and it is crucial to recognize that it assumes linear relationships. This constraint may result in consistent discrepancies between predictions and observations, particularly in cases where wear behavior displays intricate nonlinearities. The distribution and patterns of residuals (difference between observed and predicted values) were examined to evaluate this limitation. Heteroscedasticity or significant residual structure would imply nonlinear effects that LR is unable to capture.

#### K-Nearest neighbors regression

The K-nearest Neighbors regression (KNNR) technique is a widely used nonparametric algorithm known for its low error rate and effective error distribution. The KNN algorithm typically relies on calculating the Euclidean distance (qi) between a test sample and selected training samples^48^ (Eq. 2).

2 $$\:{q}_{i}=\sqrt{\sum\:_{j=1}^{p}({x}_{j}-{x}_{ij}{)}^{2}}$$

where $\:{q}_{i}$represents the distance between the test point and the $\:{i}^{th}$training sample, $\:p$is the total number of features, and $\:{x}_{ij}$is the value of the $\:{j}^{th}$feature for the $\:{i}^{th}$sample.

Let the distances between the current input sample and its $\:k$ nearest neighbors be denoted by $\:{q}_{i}$ $\:(i=1,\dots\:,k)$. The corresponding observed or predicted wear values of these neighbors are represented by $\:{V}_{i}$. The predicted wear value $\:\widehat{V}$ for the input sample is then computed as a weighted average of the neighbor values (Eq. 3):

3 $$\:\widehat{V}=\:\frac{\sum\:_{i=1}^{k}wiVi}{\sum\:_{i=1}^{k}wi}$$

where $\:{w}_{i}=1/{q}_{i}$assigns higher weights to neighbors closer to the test sample, thereby improving prediction sensitivity to relevant patterns.

K-Nearest Neighbors Regression averages the wear values of the K nearest data points (neighbors) in the feature space to predict the 3-BAW. Based on experimental parameters like filler content, load, and abrading distance, distance metrics typically Euclidean distance are used to determine the training data nearest neighbors. K, the number of neighbors, is chosen to strike a balance between noise sensitivity and prediction accuracy. It operates by identifying the *k* most similar historical data sets referred to as the nearest neighbors—based on a defined distance metric. To enhance prediction accuracy, the KNN algorithm assigns higher weights to neighbors that are closer to the input data, while assigning lower weights to those that are farther away. This weighted approach ensures that predictions are more strongly influenced by the most relevant historical patterns^49,50^. Compared to linear models, this approach improves prediction accuracy by efficiently capturing local variations and nonlinear relationships between experimental variables and wear responses.

Cross-validation is essential for optimizing the choice of K because a K that is too small can cause the model to overfit (be sensitive to noise), while a K that is too large can cause the predictions to become over smooth (underfit). Furthermore, if features have different scales or levels of relevance, choosing a distance metric (beyond Euclidean, like Manhattan or Minkowski) can affect the local neighbourhood structure and prediction accuracy. In contrast to linear models, testing a variety of metrics and K values through thorough validation confirms that the model configuration optimizes predictive accuracy for the dataset, successfully capturing nonlinear and local variations in wear behavior.

#### Artificial neural network regression

The Artificial Neural Network Regression (ANNR) uses a multilayer architecture with an input layer, one or more hidden layers, and an output layer to model nonlinear relationships between input variables and output wear loss^51,52^. The network architecture used in this study included a hidden layer with 10 neurons using the ReLU activation function, an input layer that received experimental parameters like filler content, applied load, and abrading distance, and a single output neuron that represented the expected wear loss.

The Adam optimizer was used for training, with a learning rate of 0.001 spread across 100 epochs. Reducing the mean squared error (MSE) served as the foundation for the convergence criterion. About X minutes were spent training; please specify based on experimental or typical results. By tracking declining MSE trends over epochs, the model showed stable convergence.

To evaluate the generalizability of the model and avoid overfitting, an 80:20 train-test split was used for validation, along with an extra 10-fold cross-validation. With R^2^ = 0.8391 on the test set, the ANN demonstrated strong predictive accuracy and a good fit to nonlinear wear behaviours.

Reproducibility is improved by this thorough ANN configuration and validation approach, which also offers lucid insights into how training parameters and network design decisions affect precise abrasive wear prediction in hybrid composites. In this study, ANNR was utilized to predict 3-BAW based on variables such as filler content, normal load, and abrading distance. The network architecture typically consists of an input layer, one or more hidden layers with movable neurons, and an output layer that represents the expected wear.

#### Gradient boosting regression

By dividing the dataset into ten subsets and training and validating the model successively, 10-fold cross-validation was used to evaluate the robustness of the GBR model. By averaging performance across various training-testing splits, this method decreased overfitting and produced accurate generalization estimates. To ensure ideal model tuning, grid search was used to choose and validate hyperparameters like learning rate, number of boosting stages, and maximum tree depth. The model’s robustness in predicting abrasive wear behavior was confirmed by the generalized performance metrics that were produced, such as average R^2^ and RMSE across folds, which showed consistent predictive accuracy and stability.

Gradient Boosting Regression (GBR) is an ensemble machine learning technique that combines several weak learners, usually decision trees, to gradually create a strong predictive model. To minimize a given loss function, like mean squared error, the model is iteratively trained^53,54^.

Formally, the GBR starts with an initial prediction $\:{F}_{0}\left(x\right)$, often the mean of the target values. At each iteration $\:m=1,\dots\:,M$, the model computes the pseudo-residuals $\:{r}_{im}$, which are the negative gradients of the loss function with respect to the current model predictions $\:{F}_{m-1}\left({x}_{i}\right)(\text{E}\text{q}.\:4)$:

4 $$\:{r}_{im}=-{\left[\frac{\partial\:L({y}_{i},F({x}_{i}\left)\right)}{\partial\:F\left({x}_{i}\right)}\right]}_{F\left(x\right)={F}_{m-1}\left(x\right)}$$

A weak learner $\:{h}_{m}\left(x\right)$(e.g., a decision tree) is then fit to these residuals. The prediction is updated by adding a scaled version of this learner (Eq. 5):

5 $$\:{F}_{m}\left(x\right)={F}_{m-1}\left(x\right)+\eta\:\cdot\:{h}_{m}\left(x\right)$$

where $\:\eta\:$is the learning rate controlling the contribution of each learner. This procedure is repeated for $\:M$iterations, progressively refining the model by focusing on errors made by prior learners.

Previous studies have confirmed that GBR can accurately predict wear loss in composite materials. By using an iterative residual fitting procedure, GBR will certainly accurately represent intricate, nonlinear relationships in the behavior of abrasive wear^55^.

#### Random forest regression

A popular supervised ML algorithm for classification and regression problems, Random Forest (RF) Regression is renowned for its accuracy and resilience. It functions by building numerous decision trees during training, each of which is constructed using distinct random subsets of the data—a technique called bootstrap aggregating or bagging. In regression, the average of the predictions made by each tree determines the result, whereas in classification, the majority vote among these trees determines the outcome.

RF’s main benefit is its capacity to lessen overfitting, a problem that single decision trees frequently encounter. To accomplish this, each tree is trained using a random subset of features and random data samples, making sure that the trees are diverse and uncorrelated. Additionally, RF offers information on feature importance, which aids in determining which variables have the biggest impact on the predictions. To maximize model performance, hyperparameters like the number of trees (n_estimators), the maximum depth of the trees, and the maximum features taken into consideration for splits are adjusted in practice. The model is well-suited for complicated problems like forecasting abrasive wear in composite materials because of its resilience to missing data and capacity to manage high-dimensional datasets.

Using the set of experimental data with input parameters (x) likely filler loading, abrading distance, and applied normal load output parameter (y) as wear loss D = (x, y), bootstrap methods are used to find new training data sets for each tree.

Next, each node’s most important characteristics are identified. The correctness of the method is assessed by calculating the set of predictable values for test data after the trees have been created. Equation 6 is the model that serves as the foundation for linear regression.

6 $$\:f\left(X\right)={\beta\:}_{0}+\sum\:_{j=1}^{p}{X}_{j}{\beta\:}_{j}$$

Conversely, regression trees are predicated on a model of the type Eq. 7.

7 $$\:f\left(X\right)=\sum\:_{m=1}^{M}{C}_{m}{.1\left(X\epsilon{\:R}_{m}\right)}_{\:}$$

where $\:{\:R}_{1}$------$\:{\:R}_{m}$ denotes a feature space division^56^.

#### eXtreme gradient boosting

Based on the idea of gradient boosting with decision trees, eXtreme Gradient Boosting (XGBoost) is a very sophisticated and effective machine learning algorithm. By gradually constructing an ensemble of trees, each of which attempts to correct the residual errors of the one before it, it is excellent at managing non-linear relationships among input variables and iteratively increasing the accuracy of the model. Faster tree construction and a unique tree search algorithm are two significant improvements over conventional gradient boosting that make XGBoost unique.

One of XGBoost’s main characteristics is the regularization term it uses in the loss function to penalize overly complex trees, which helps control model complexity and avoid overfitting^57^. Strong predictive performance is achieved by combining this regularization with the iterative error correction of boosting. For instance, Ferdousi et al.^58^ used XGBoost to model the properties of composites and reported a prediction accuracy with a R^2^ value of 98.8%. To improve model generalization and effectively manage overfitting, XGBoost is essentially an optimized version of Gradient Boosting Machines (GBM) with extra regularization to determine the maximum gain at each node during tree building.

## Results and discussion

### FTIR of µCC-modified H/B F-Ep hybrid composites

**Fig. 3:**
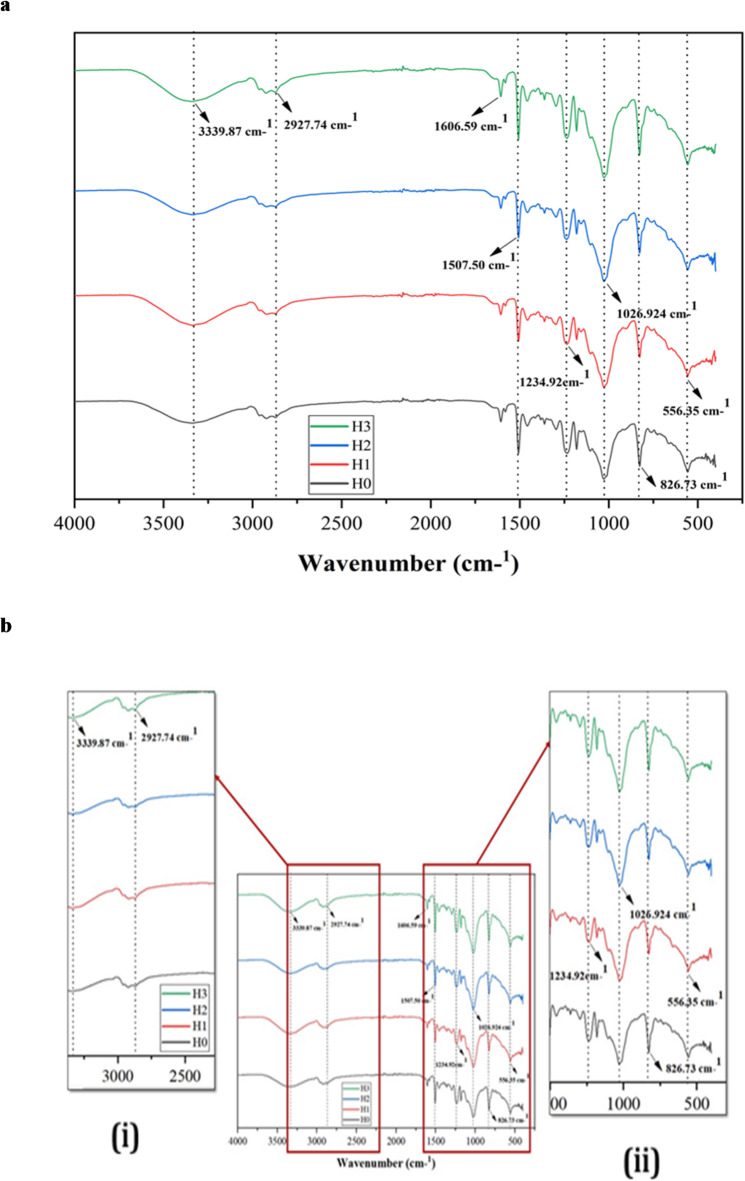
(**a**) FTIR data of µCC-modified H/B F-Ep hybrid composites. (**b**) FTIR spectrum of µCC-modified H/B F-Ep hybrid composites; inset is zoomed spectrum (i) 3200–3600 cm^− 1^, and (ii) 1000–1300 cm^− 1^.

The Fourier transform infrared (FTIR) spectra of the hemp/bamboo fabric–epoxy (H/B F–Ep) composites with increasing microcrystalline cellulose (µCC) content (H0–H3) are presented in Fig. 3a, illustrating the effect of µCC incorporation on the functional group interactions within the composite matrix. With insets offering a close-up of two distinct spectral regions, (i) the 3200–3600 cm⁻¹ range, which emphasizes hydroxyl group (–OH) stretching vibrations, and (ii) the 1000–1300 cm⁻¹ range, which emphasizes the C–O and C–O–C stretching vibrations linked to cellulose and epoxy matrix components, their corresponding FTIR spectra are displayed in Fig. 3b. As µCC loading rises, the broad O–H stretching band seen at 3339 cm⁻¹ gets stronger, suggesting that cellulose incorporation has increased the abundance of hydroxyl groups. Higher hydrogen bonding potential within the composite matrix and at the fiber–epoxy interface is confirmed by this improvement, which is in line with earlier reports for cellulose-reinforced epoxy systems^59^.

A notable increase in intensity is observed in the C–H stretching bands around 2927 cm⁻¹, which indicates changes in the polymer backbone environment and validates interactions between the epoxy matrix, natural fibers, and µCC. Changes in the epoxy–cellulose and natural fiber–matrix bonding are linked to variations in the C = O stretching region, which is located between 1507 and 1606 cm⁻¹. Like the patterns noted for hybrid cellulose/epoxy composites and other natural fiber–epoxy hybrids, this is ascribed to expanded hydrogen bonding and aromatic group dynamics^60–62^. At higher µCC contents, additional absorption bands around 1234, 1027, and 826 cm⁻¹ become more noticeable, indicating successful cellulose incorporation and changed matrix chemistry. According to recent FTIR studies on hybrid composites, these bands correlate to C–O–C stretching and other vibrational modes unique to cellulose. Improved interfacial bonding is confirmed by peak shifts and intensity changes, which is in line with findings for cellulose/epoxy and coir/glass fiber/epoxy systems^63^.

**Table 2:** Observed FTIR spectral changes with increasing µCC loading.

Composites	Key FTIR Peaks (cm^-1^)	Main changes with µCC addition
H0	1234, 1027, 856, 826	Baseline; characteristic of epoxy and fibers
H1	3339, 2927, 1600	O-H and C-H peaks increase; aliphatic C–H stretching vibrations
H2	3339, 2927, 1507, 1027	More intense O-H bands; cellulose peaks prominent
H3	3339, 2927, 1600, 1235	Strongest cellulose features; peak shifts

Observed FTIR spectral changes with increasing µCC content are summarized in Table 2. Consistent with the observations in H/B F-Ep composites, similar results from the literature on FTIR spectra of cellulose-filled Ep composites typically report spectral changes as the µCC content increases. The broad O–H stretching band around 3300–3450 cm⁻¹ usually becomes more noticeable as the cellulose content rises, according to FTIR studies of cellulose-Ep composites. This suggests that cellulose contributes more hydroxyl groups to the mixture^64^. This implies that the composite has a higher potential for hydrogen bonding. Concurrently, changes in aromatic C = C stretching region (1600 –1500 cm^−^¹) indicate shifting interactions between the epoxy matrix, cellulose, and natural fibers; these changes frequently indicate expanded hydrogen bonding or other interfacial interactions that affect the polymer network^65^. Furthermore, as cellulose is added, modifications in the C–H stretching bands around 2900–2930 cm^−^¹ indicate changes in the polymer backbone environment^66^. All these FTIR spectral changes show how adding more cellulose improves interfacial bonding and alters the epoxy composite’s chemical environment, both of which can eventually result in better composite performance.

In summary, the FTIR spectral changes, which range from increased O–H intensity to recognizable cellulose-specific bands, show stronger hydrogen bonding and altered interactions among cellulose, fibers, and matrix as the µCC content increases. Similar FTIR results in the literature support these effects, which lead to better chemical compatibility and correlate well with enhanced composite properties.

### Hardness

**Fig. 4:**
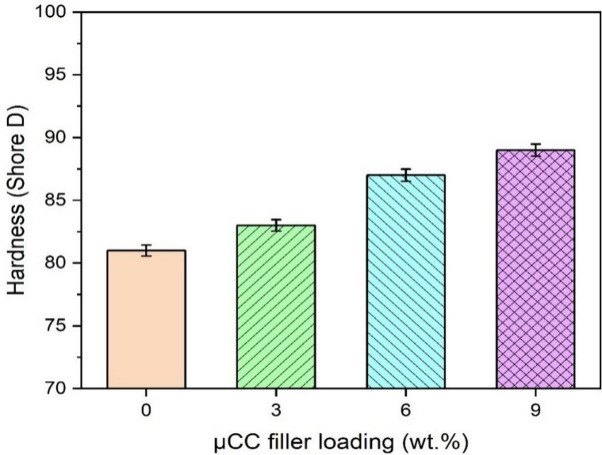
Hardness of µCC-modified H/B F-Ep hybrid composites.

The Shore D hardness of the microcrystalline (µCC) modified H/B F-Ep composite system is shown in Fig. 4. Hardness gradually increases as filler content rises, going from 81 (0 wt% µCC) to 89 (9 wt% µCC) improvement. The rigid, reinforcing effect of µCC particles, which improve resistance to surface deformation and effectively distribute applied loads within the matrix, is reflected in this trend. The sustained rise in µCC up to 9 wt% in this study indicates strong interfacial bonding and good filler dispersion, underscoring the successful incorporation of µCC into the composite matrix.

Recent literature has reported similar findings. For example, Bandaru et al.^66^ showed that by strengthening the matrix and enhancing fiber-matrix adhesion, µCC fillers greatly increase the mechanical strength of flax/Ep composites, including improvements in hardness. The addition of µCC to polypropylene (PP) composites improves hardness because compatibilizers improve fiber-matrix interaction, according to Lupidi et al.^67^. Another study by Rathnayake et al.^68^ highlighted the reinforcing potential of well-dispersed cellulose fillers by reporting increases in tensile and hardness properties of up to 8 wt% in PP composites reinforced with 5 wt% µCC.

Improved surface hardness is typically ascribed to increased rigidity and efficient load transfer from the filler to the epoxy matrix in conjunction with consistent dispersion and robust interfacial bonding as the µCC content rises. More filler content, however, can cause particle agglomeration after a certain ideal filler threshold, which is usually between 8 and 10 wt% for cellulose-based composites, according to several studies. Agglomeration can lead to weak spots or microstructural flaws, interfere with even stress distribution, and prevent further improvements in mechanical qualities like durability. This phenomenon happens because too much filler tends to group together instead of spreading evenly, which damages the filler–matrix interface and may lower reinforcement effectiveness^69^. Thus, even though the filler content keeps increasing, the observed tendency for hardness to plateau at 9 wt% µCC in the current study probably represents the start of filler agglomeration, which restricts further mechanical improvement. Recent reports on composites utilizing cellulose microcrystals and nanocrystals, where optimal performance and property gains are consistently observed below the threshold for noticeable aggregation^70–72^, strongly support this interpretation.

Through improved interfacial bonding and load transfer mechanisms, these studies demonstrate that cellulose micro and nanocrystals function as efficient reinforcements in thermosetting and thermoplastic matrices, increasing hardness. In summary, even though the composite system exhibits strong hardness increases up to 9 wt% µCC, adding more filler after this point may cause agglomeration and less effective reinforcement, which would explain the observed plateau in property enhancement.

### Applied load, abrading distance and µCC loading on wear loss of H/B F-Ep composites

The wear loss of textile hybrid composites reinforced with different weight percentages of microcrystalline cellulose (µCC) filler into H/B F-Ep composites are listed in Table 3. Wear tests were conducted under varying abrading distances, applied loads, and µCC loadings according to a Taguchi L16 orthogonal array design. The resulting wear losses were measured, and signal-to-noise (S/N) ratios were calculated using the “smaller-the-better” criterion to assess wear minimization performance. Wear resistance is shown by the SN ratio, where reduced wear loss is associated with larger values (Table 3). Under the mildest test conditions of 250 m abrading distance and 5 N load, Test Run 1 (H0 composite with 0 wt% µCC) showed the lowest wear loss of any sample, at 0.0804 g. This implies that the base composite functions effectively under low stress circumstances.

**Table 3:** List the wear loss of µCC-modified H/B-F/Ep composites as per L16 orthogonal array.

Test Run	µCC (wt%)	Abrading Distance (m)	Load (*N*)	Wear loss (g)	SN ratios (dB)
1.	0	250	5	0.0804	21.8949
2.	0	500	10	0.2188	13.1991
3.	0	750	15	0.5029	5.9704
4.	0	1000	20	1.0253	-0.217
5.	3	250	10	0.0984	20.1401
6.	3	500	5	0.1188	18.5015
7.	3	750	20	0.7254	2.7885
8.	3	1000	15	0.5230	5.6299
9.	6	250	15	0.1062	19.4775
10.	6	500	20	0.3508	9.0988
11.	6	750	5	0.1896	14.4419
12.	6	1000	10	0.3446	9.2537
13.	9	250	20	0.0976	20.2110
14.	9	500	15	0.1366	17.2910
15.	9	750	10	0.2517	11.9823
16.	9	1000	5	0.2024	13.8758

In contrast, the H3 sample with 9 wt% µCC (Test Run 13) showed the least amount of wear loss (0.0976) at a short abrading distance of 250 m, and a relatively high load of 20 N when examining the µCC-reinforced H/B F-Ep composites. This suggests that µCC can greatly improve wear resistance, particularly in more demanding situations. Although H1 and H2 (3 wt% and 6 wt% µCC) demonstrated only modest gains, H3 continuously outperformed, indicating that a larger µCC concentration enhances the longevity of the composite. Overall, H3 performed better in more realistic or demanding testing scenarios, even though the unfilled composite had the best wear resistance under light use conditions.

**Fig. 5:**
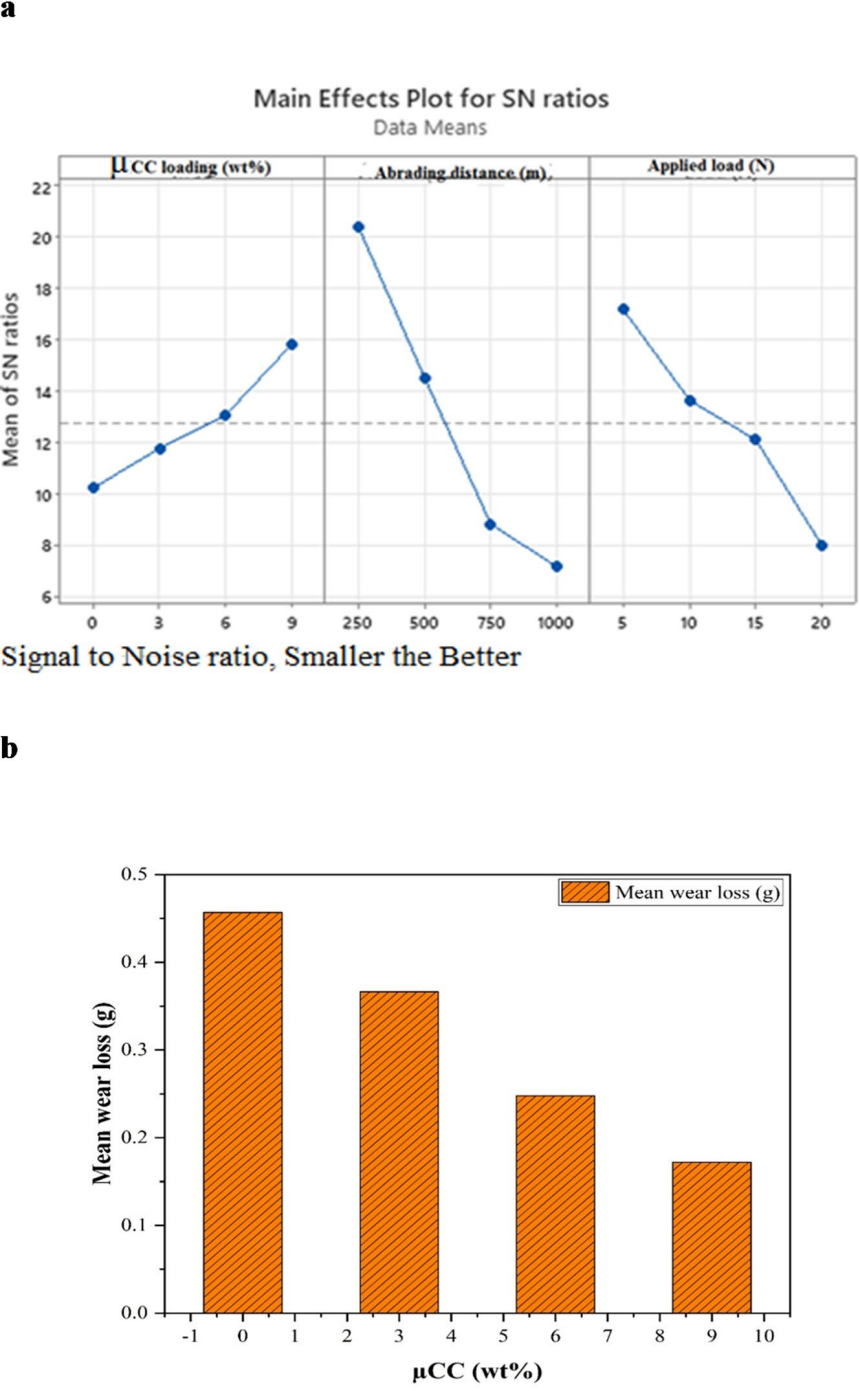
(**a**) Main effects plot showing the influence of µCC loading, abrading distance, and applied load on SN ratios for the wear performance of H/B F-Ep hybrid composites. (**b**) Mean wear loss as a function of µCC content.

Figure 5a shows the primary impacts of applied load, abrading distance, and µCC loading on the SN ratios, which represent the composites’ wear resistance. In line with other research on ceramic and mineral-filled composites, an upward trend in SN ratios with increasing µCC concentration indicates that greater filler levels improve wear resistance by increasing hardness and load-bearing capacity^73,74^. As the abrading distance increases from 250 m to 1000 m, however, SN ratios drastically decrease, suggesting that extended exposure to abrasive conditions speeds up surface degradation^75^. In a similar vein, raising the applied load causes SN ratios to decrease, which indicates more material removal because of increased heat generation and frictional forces. This phenomenon is frequently seen in polymer matrix composites^76^.

Figure 5b shows the mean wear loss trend as a function of µCC (wt%). In line with the statistical interpretation and ranking discussion, this figure unequivocally demonstrates a constant drop in mean wear loss as µCC content (0–9 wt%) increases, indicating that increased µCC loading improves the composite’s wear resistance. Although FTIR research demonstrates that µCC greatly improves material hardness and bonding, its lower delta and rank indicate a reduced proportional influence on wear performance when compared to the more important mechanical parameters like load and abrading distance. Nonetheless, it still plays a significant part in enhancing the overall durability of composites.

These findings support earlier research suggesting that a higher filler content (e.g., µCC) improves wear resistance by improving surface hardness and load-bearing capacity^74^. Furthermore, the detrimental effect of larger loads and longer abrading distances on SN ratios is in line with tribological principles, which state that wear degradation is accelerated by increased mechanical stress^75,76^.

Table 4 summarizes the average wear loss at different applied load, abrading distance, and µCC content levels. It displays the response table for means. Abrading distance has the highest delta value (0.42818) of the three control parameters, indicating that it has the most impact on wear performance. Load (Δ = 0.40196) and µCC content (Δ = 0.28478) come next.

Although µCC is essential for increasing the composite’s hardness and FTIR properties, its rank = 3 and delta = 0.28478 show that its direct statistical impact on wear loss is less significant than that of load and distance affecting the mechanical properties. This is because the contact mechanics and frictional energy dissipation during wear are directly governed by the abrading distance and load, which results in a greater variation in wear loss. On the other hand, µCC mainly helps by strengthening the microstructure, which results in a slow but consistent increase in wear resistance.

Higher µCC content improves wear resistance, as evidenced by the constant decrease in mean wear loss as µCC loading increases from 0% to 9 wt%. Plotting mean wear loss versus µCC percentage shows a monotonic drop, which visually validates this tendency. Statistically speaking, the degrees of freedom (DF = 3) represent the number of independent comparisons available for evaluating the factor’s impact on wear loss and correspond to the four µCC levels (0, 3, 6, 9 wt%) minus one.

**Table 4:** Response table for Means.

Level	µCC (wt%)	Abrading distance (m)	Load (*N*)
1	0.45685	0.09565	0.14782
2	0.36641	0.20626	0.22837
3	0.24781	0.41741	0.31718
4	0.17208	0.52383	0.54978
Delta	0.28478	0.42818	0.40196
Rank	3	1	2

**Table 5:** Analysis of Variance.

Source	DF	Adj SS	Adj MS	F-Value	*P*-Value	% of Contribution
µCC (wt%)	3	0.19054	0.063514	7.66	0.018	18.006
Abrading Distance (m)	3	0.45585	0.151951	18.33	0.002	43.079
Load (N)	3	0.36203	0.120677	14.55	0.004	34.212
Error	6	0.04975	0.008292			4.701
Total	15	1.05818				100

S: 0.0911, R-sq: 95.30%, R-sq (adj): 88.25%, R-sq (pred): 84.32%.

Table 5 displays the results of the Analysis of Variance (ANOVA), which statistically quantify the individual and comparative effects of applied load, abrading distance, and µCC content on wear loss. With p-values less than 0.05, all three factors show statistically significant effects (µCC content: *p* = 0.018; abrading distance: *p* = 0.002; load: *p* = 0.004). The dominant factors are abrading distance (F = 18.33), load (F = 14.55), and µCC loading (F = 7.66), according to the F-values.

Physically, cumulative material removal during the abrasion process is the main cause of abrading distance; the longer the distance, the higher the exposure and resulting wear^77^. Through increased deformation and micro-cutting mechanisms, load accelerates wear by increasing the contact pressure between the abrasive and composite surface^78^. By increasing hardness and load-bearing capacity, the µCC filler content reduces material loss and mitigates the abrasive impact, improving the composite’s resistance to wear. These elements work together to explain why increasing µCC loading while reducing load and abrading distance can maximize abrasion resistance.

**Fig. 6:**
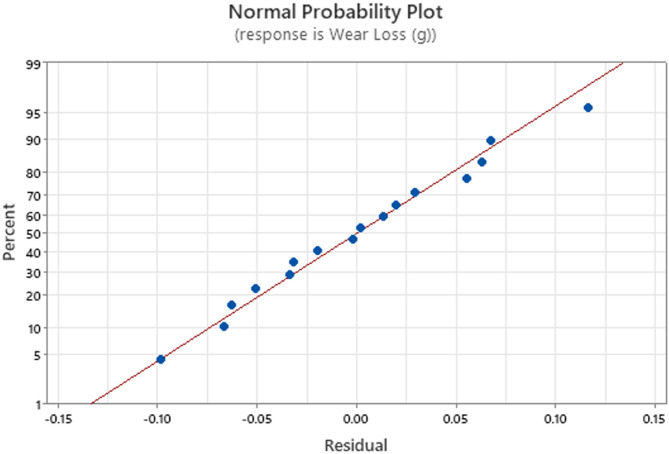
Normal probability plot of response to the wear loss.

The residual distribution for the wear loss data is displayed in the normal probability plot in Fig. 6 to determine if it follows a normal distribution. A theoretical normal distribution is represented by a straight reference line, which is plotted against the residuals. It is clear from the data points’ close alignment with this line that the residuals are approximately normally distributed. The premise of normalcy appears to be met because there are no notable outliers or deviations. This validates the use of statistical tools like ANOVA by confirming that the model used to evaluate wear loss is reliable and statistically sound.

#### Wear-out surface morphology

**Fig. 7:**
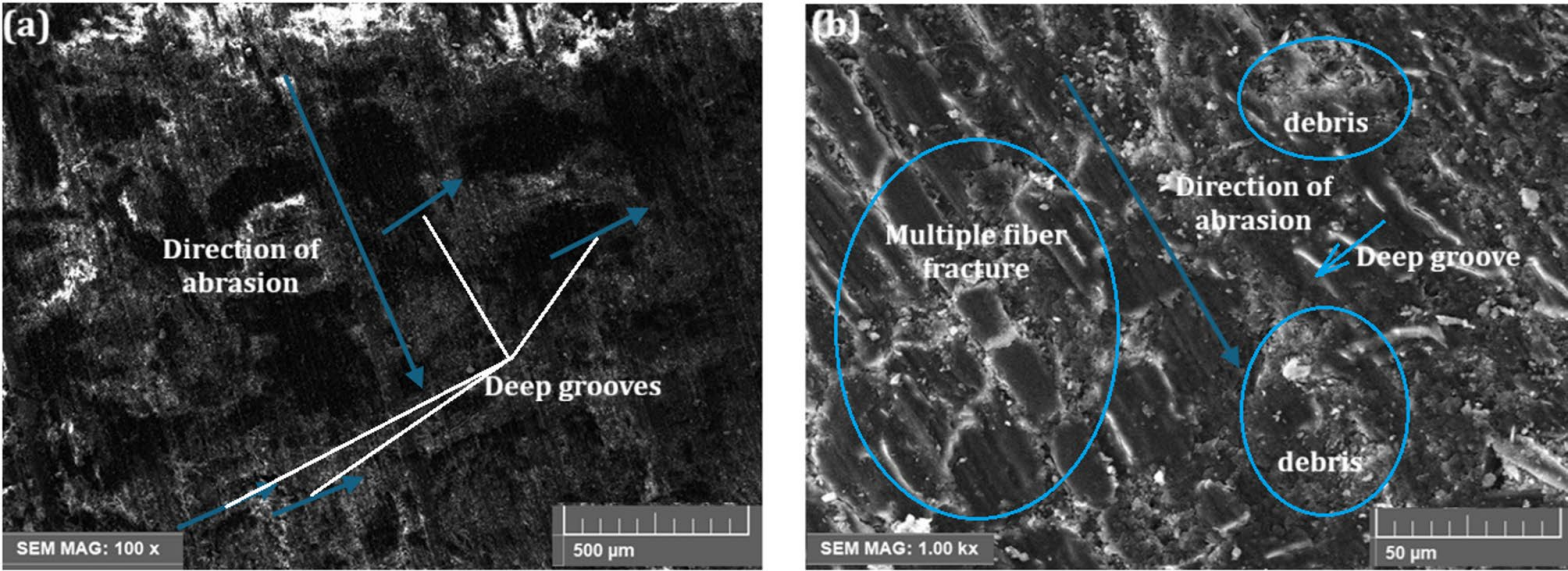
(**a**,** b**) Worn surface morphologies of H/B F–Ep composites: (**a**) At 10 N and 500 m abrasion, SEM (100×) reveals pronounced deep grooves along the abrasion direction, highlighting severe two-body wear. (**b**) Higher magnification (1000×) shows multiple fiber fractures and distinct matrix debris, marking poor fiber–matrix adhesion and brittle failures. All major features are clearly labelled for direct interpretation.

The hemp-bamboo fiber epoxy (H/B F-Ep) composites’ worn surface morphologies exhibit significant distinctions based on the testing conditions and the presence of µCC fillers. Figure 7a, b presents the unfilled H/B F-Ep composite after 500 m abrasion at 10 N load, revealing severe two-body abrasion characterized by deep grooves, cut fibers, matrix debris, and fiber fractures. These characteristics suggest brittle fiber failure, insufficient load transfer, and poor fiber-matrix adhesion, all of which contribute to significant material loss. Aggressive abrasive interactions that are primarily associated with silica sand particles are confirmed by the deep grooves and fiber cuts that are oriented transversely to the abrading direction. Wear is accelerated by micro-cutting and unstable debris generation, which is suggested by the dense matrix debris and premature epoxy separation.

**Fig. 8:**
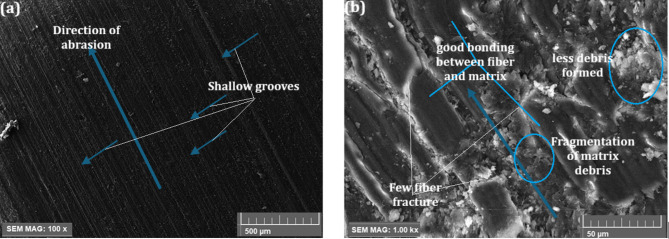
(**a**,** b**) Worn surface morphologies of 9 wt% µCC-filled H/B F–Ep composite: (**a**) At 5 N load and 1000 m abrasion, SEM (100×) reveals shallow grooves parallel to the abrasion direction, indicating reduced wear severity. (**b**) Higher magnification (1000×) shows good bonding between fiber and matrix, few fiber fractures, less debris formation, and fragmented matrix debris.

On the other hand, under 5 N and 1000 m abrasion, the 9 wt% µCC -filled H/B F-Ep composite, as seen in Fig. 8a, b, shows noticeably milder wear patterns. The predominant wear mechanism here shifts from severe 2-body abrasion to a combination of 3-body abrasion, debris compaction, and crack deflection due to stepped fiber wear, shallow grooves, and localized microcracks, all of which indicate improved interfacial bonding. Through processes like energy dissipation and crack bridging, the µCC particles function as micro-bearings inside a layer of compacted debris, minimizing direct asperity-fiber contact and averting catastrophic fiber failures. Better wear resistance and surface integrity are a result of decreased fiber pull-out and improved load-sharing between fibers and matrix. These microstructural findings are consistent with the reduced wear loss shown in Fig. 5; Table 3.

Figures 7 and 8 demonstrate that the microstructural and mechanical performance of H/B F-Ep composites are significantly enhanced by an increase in µCC content. Better chemical bonding at the interfaces was confirmed by FTIR analysis, which revealed intensified hydroxyl (O–H) and carbonyl (C = O) peaks with greater µCC loading. This is demonstrated by the hardness results, which show improved resistance to surface deformation as Shore D values increased steadily with µCC addition. These results are further corroborated by the worn surface morphologies. Figure 7 showed that unfilled composites experience severe two-body abrasion, which is indicative of weak fiber–matrix adhesion and is characterized by deep grooves, numerous fiber fractures, and extensive debris. Conversely, Fig. 8 demonstrates that µCC-filled composites have stronger bonding between fiber and matrix, fewer fiber fractures, shallower grooves, and less debris formation. These characteristics align with a shift toward improved crack deflection, matrix debris compaction, and softer three-body abrasion.

Wear loss values decreased in tandem with an increase in µCC content, indicating a positive change in wear mechanisms and an improvement in composite hardness and interfacial adhesion. Overall, the improved abrasion performance seen in the µCC-reinforced composites is made possible by improved chemical bonding enhancements identified by FTIR, which are closely associated with increased mechanical properties and wear resistance.

#### Correlation of wear resistance with FTIR, hardness, and the Lancaster-Ratner model

For hemp/bamboo hybrid fabric–epoxy composites, the reinforcing effect of microcrystalline cellulose (µCC) causes a quantitative correlation between hardness and FTIR data and the wear mechanism shift from severe 2-body abrasion to milder 3-body abrasion. Stronger hydroxyl (O–H) and carbonyl (C = O) peaks in FTIR are indicative of increased interfacial bonding through hydrogen bonding between µCC, the natural fibers, and the epoxy matrix as the µCC content rises. Structural integrity and load transfer are enhanced by this superior chemical interaction. In tandem, Shore D hardness increases steadily with µCC loading, indicating a greater ability of the surface to withstand deformation. Because abrasion resistance scales inversely with hardness, these hardness improvements directly result in lower wear rates. This is corroborated by morphological wear surface analysis, which indicates that as µCC increases, the debris compaction, crack deflection, and stepped fiber wear typical of 3-body abrasion replace the deep grooves and fiber fracture characteristic of 2-body abrasion.

The Lancaster-Ratner theoretical framework links better interfacial bonding and higher hardness with less material removal by showing that the wear rate is inversely related to the product of material strength and strain to break. It is confirmed that µCC plays a quantitative role in strengthening the composite and changing the wear mechanism from 2-body to 3-body abrasion when the model’s predicted wear resistance trends match experimental observations. Thus, the Lancaster-Ratner model quantitatively links wear resistance improvements and mechanistic wear shifts to hardness measurements that quantify mechanical robustness and FTIR chemical changes that quantify bonding enhancements. This thorough quantitative analysis demonstrates the way µCC plays a crucial role in enhancing composite tribological performance and promoting the creation of more robust, eco-friendly natural fiber composites.

### Machine learning model performance

**Fig. 9:**
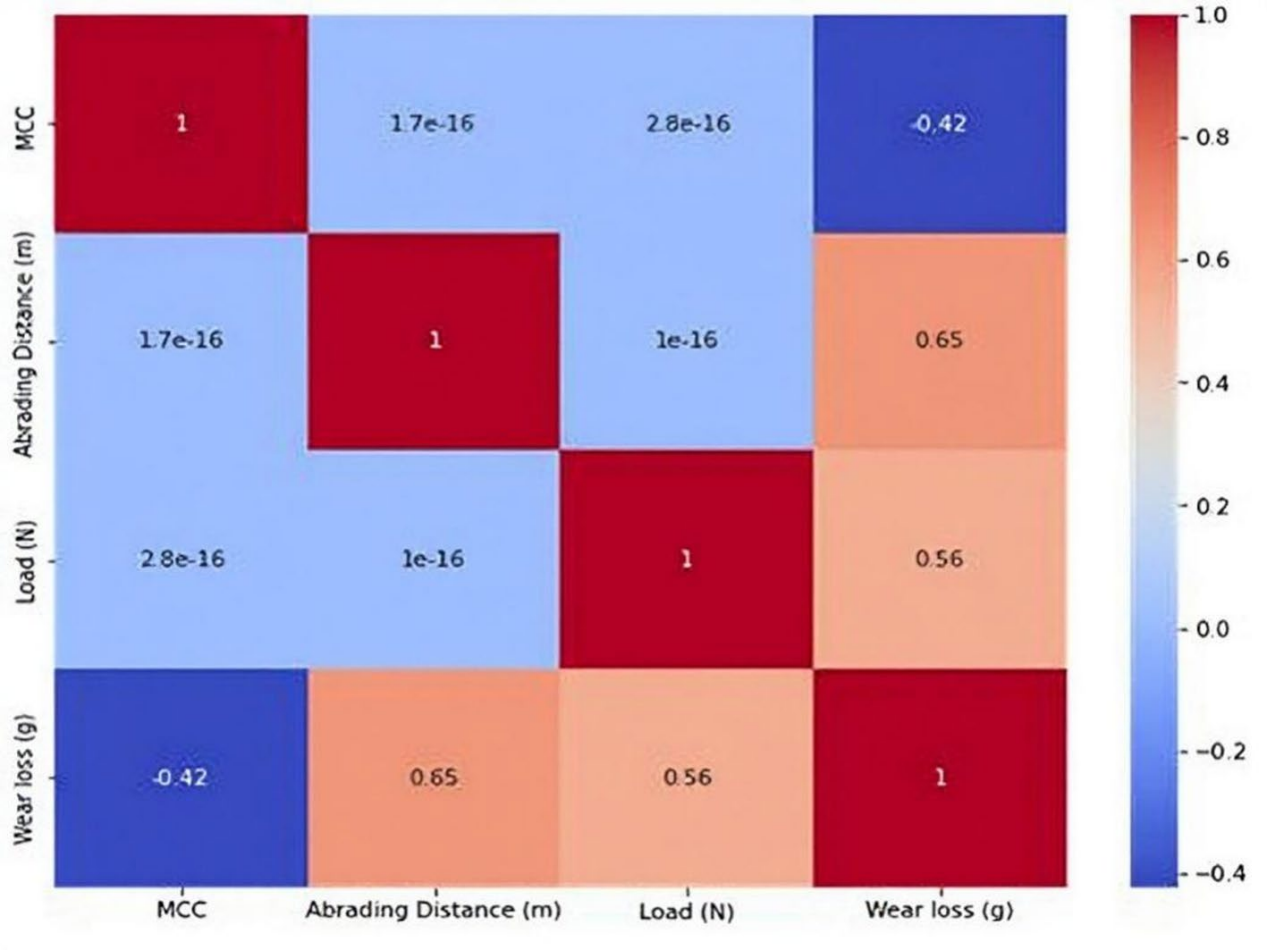
The correlation heatmap of abrasive wear loss.

The abrasive wear loss correlation heatmap in Fig. 9 illustrates distinct relationships between the parameters under study. Wear loss is almost positively correlated with both applied load (*r* = 0.56) and abrading distance (*r* = 0.65), suggesting that longer abrasion distances and greater loads significantly accelerate material removal. On the other hand, wear loss and µCC show a slightly negative association (*r* = – 0.42), indicating that a higher µCC loading increases resistance to wear. Correlations between load, abrading distance, and µCC, however, are still very weak, indicating that these factors function independently in this dataset.

These findings are consistent with accepted tribo-principles. The wear volume is inversely related to material hardness and proportionate to the product of load and abrading distance, as per Archard’s wear law^79^. As a result, the positive relationships between wear loss, load, and abrading distance that were noted in Fig. 9 are in line with theory and commonly seen in abrasive wear research. For instance, studies on polymer composites have demonstrated that wear loss increases gradually as normal load and sliding distance increase^80,81^. Research on polymer matrix composites also revealed a direct association amongst load, abrading distance, and specific wear rate, demonstrating that higher operational stresses accelerate material removal^82^. In this case, the negative relationship between wear loss and µCC suggests that µCC mitigates wear during abrasion in a way to that of hardness or strengthening characteristics.

**Fig. 10:**
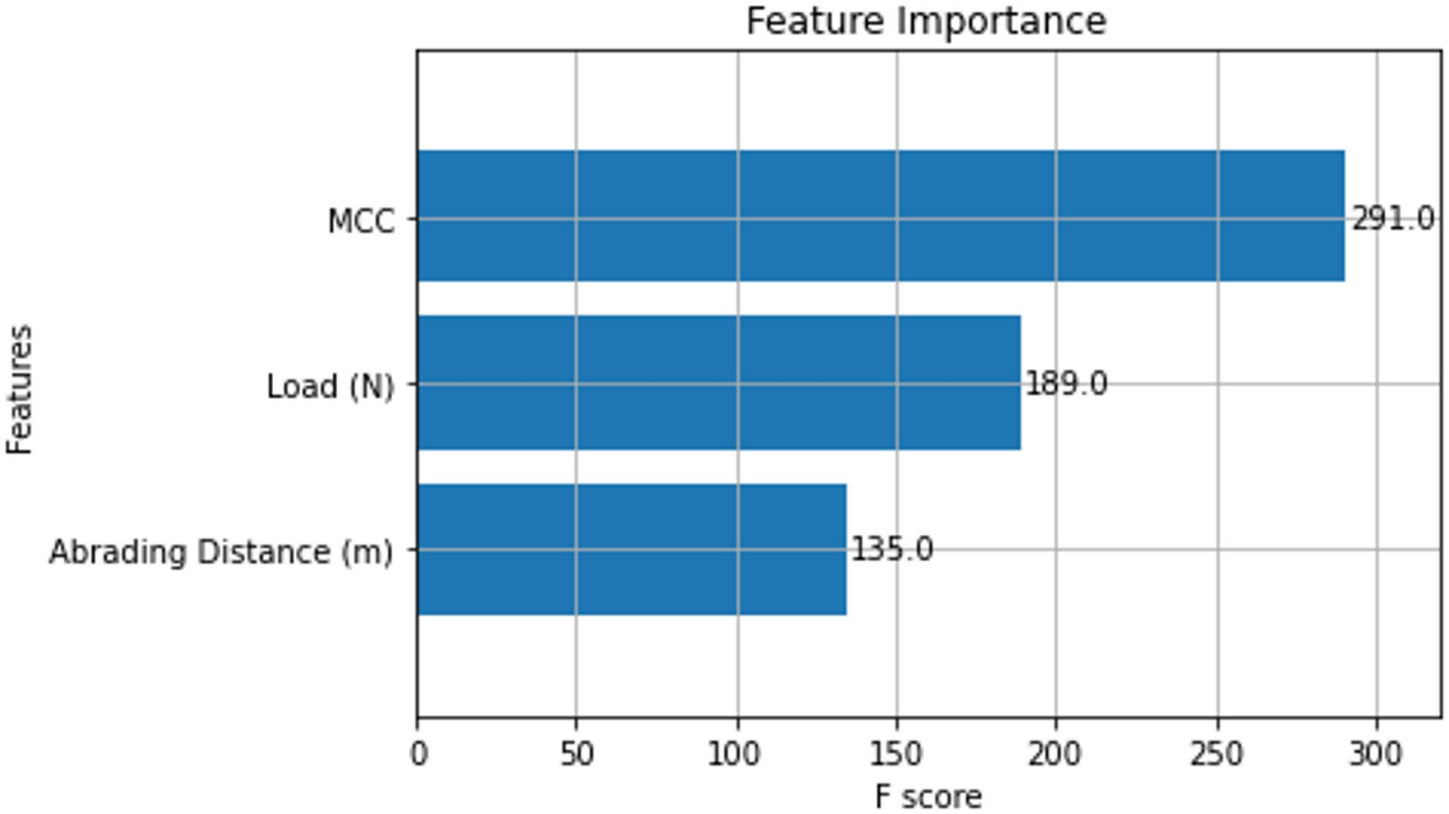
Feature importance scores for predicting 3-BAW of µCC-modified H/B F-Ep composites.

The feature importance scores obtained by applying our ML model to forecast 3-BAW of µCC-filled H/B F–Ep composites are shown in Fig. 10. The most significant factor (F-score = 291) is the µCC loading, which outweighs the abrading distance (F-score = 135) and applied load (F-score = 189). This demonstrates unequivocally that adding µCC dramatically improves the composite’s ability to withstand wear by strengthening interfacial bonding, increasing hardness, and causing microstructural alterations that reduce material loss.

The prominence of µCC is consistent with recent research by Yan et al. who showed that filler loading (sericite in epoxy coatings) held the highest feature importance for predicting wear rate and friction coefficient using gradient boosting regression. For wear rate, prediction accuracies reached 85.7%^83^. Similarly, load and speed were found to be the main determinants of wear rate in PTFE composites using ML-based tribological modeling (R^2^ = 0.91). Nevertheless, the filler loading still had a substantial impact on performance in this case^84^. The current work model’s significant applied load impact also aligns with more general patterns. By using tree-based algorithms with interpretable feature importance outputs, ML-driven analyses of functionally graded eggshell/magnesium composites revealed that applied load was the primary determinant in predicting wear rate across multiple zones, surpassing more intricate deep models^85^.

Together, the features of importance results show that the composition of the material, especially µCC loading, is a key factor in wear, even though operational parameters like load and abrading distance are crucial drivers of wear, as supported by tribological ML studies. This highlights the accuracy and usefulness of ML in recognising the key variables influencing composite wear. Echoing the growing momentum of ML-augmented materials design, such insights are helpful for forecasting performance and optimizing formulations with fewer experimentation.

#### Linear regression

**Fig. 11:**
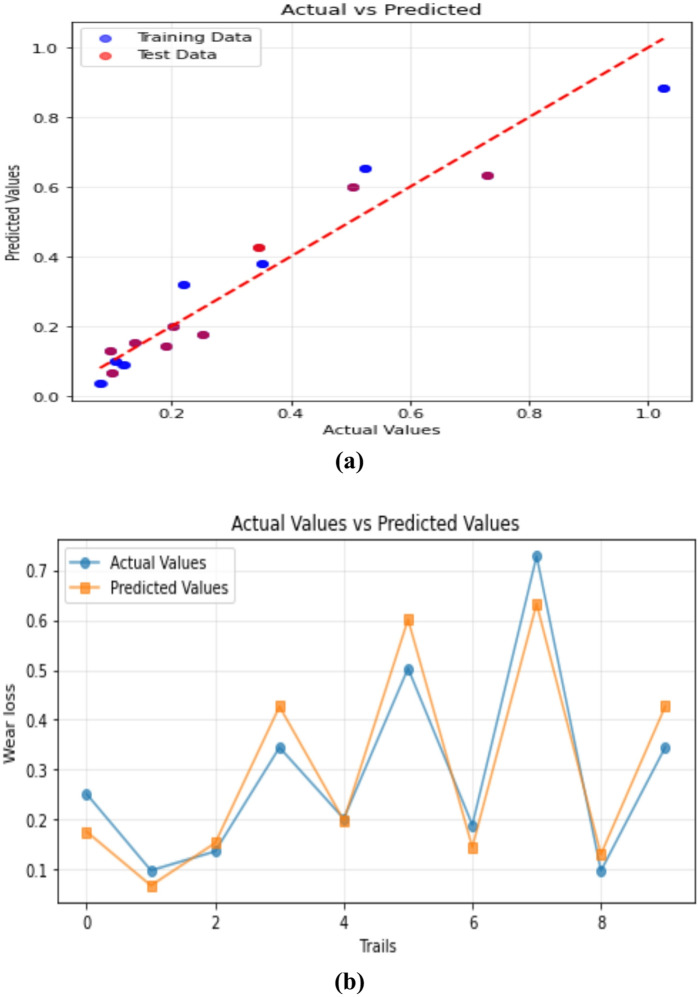
Predictive performance of the LR model for forecasting 3-BAW in µCC-filled H/B F-Ep composites: (**a**) actual vs. predicted wear loss, and (**b**) actual and predicted values are compared trial-by-trial.

The Linear Regression (LR) model’s predictive capacity for envisaging 3-BAW in µCC-filled H/B F–Ep composites are shown in Fig. 11a and b. When comparing actual and expected wear loss, the data points in Fig. 11a’s scatter plot closely resemble the diagonal line, suggesting that the LR model is reliable and in good agreement of training data and testing data (Test size = 0.2 and Random state = 42). The training and test set predictions are displayed as blue and red points, respectively, in each instance. It is evident that, for most individual tests, the predicted values deviate significantly with the experimentally measured ones. Even though the LR model effectively captured the general wear loss trend, noticeable deviations were observed due to its inability to model nonlinear tribological interactions. This limitation has been acknowledged, and LR is retained as a baseline comparison model to benchmark the performance of advanced nonlinear algorithms such as GBR, ANN, RF, and XGBoost.

The comparison of real and predicted values over several trials is further shown in Fig. 11b, which provides further evidence that the LR model accurately depicts the overall wear loss pattern with little variation. Despite being a straightforward machine learning technique, these findings demonstrate that linear regression can yield significant predictive accuracy in tribological modeling of composite materials^84,85^.

#### Gradient boosting regression

**Fig. 12:**
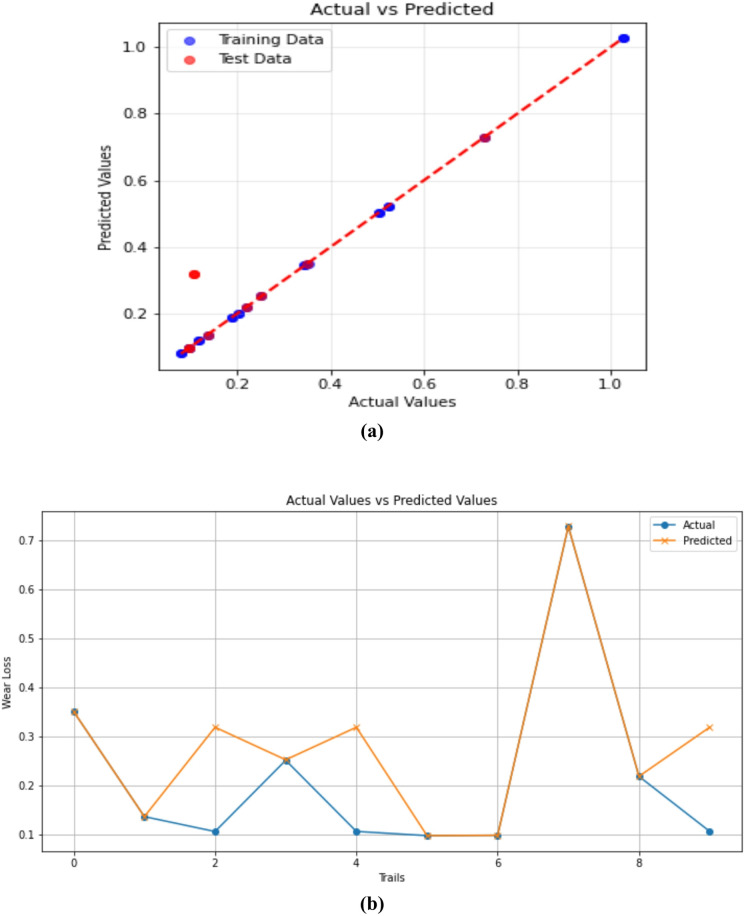
Gradient Boosting Regression predictions of 3-BAW in µCC-filled H/B F-Ep composites: (**a**) actual vs. predicted wear loss, (**b**) actual and predicted values are compared trial-by-trial.

To ensure robustness and avoid overfitting, the GBR model was trained and evaluated using 10-fold cross-validation and grid-search hyperparameter optimization (learning rate, n_estimators, and max_depth). The averaged R² and RMSE values across all folds confirmed consistent performance and strong generalization capability of the GBR model. The effectiveness of the GBR model to predict 3-BAW of µCC-filled H/B F-Ep composites are demonstrated in Fig. 12a and b. The scatter plot of actual versus anticipated wear loss in Fig. 12a demonstrates that data points closely match the diagonal, demonstrating the GBR model’s superior fitting ability and high predictive accuracy. It is evident that, for most individual tests, the predicted values closely align with the experimentally measured ones, with only minimal deviations. The robustness of the model is confirmed by Fig. 12b, which shows real and expected wear loss over trials. The predicted trend almost coincides with the experimental data. These outcomes align with recent research demonstrating GBR’s superior capacity to capture nonlinear relationships in composite materials and tribological systems^84,86,87^. By efficiently managing feature interactions and reducing mistakes, GBR outperforms Linear Regression in producing predictions, which makes it more appropriate for intricate wear modeling. The parameters considered for GBR’s are Test size = 0.2, Random state = 42, n_estimators = 200, learning rate = 0.05 and max_depth = 3.

#### Artificial neural network regression

**Fig. 13:**
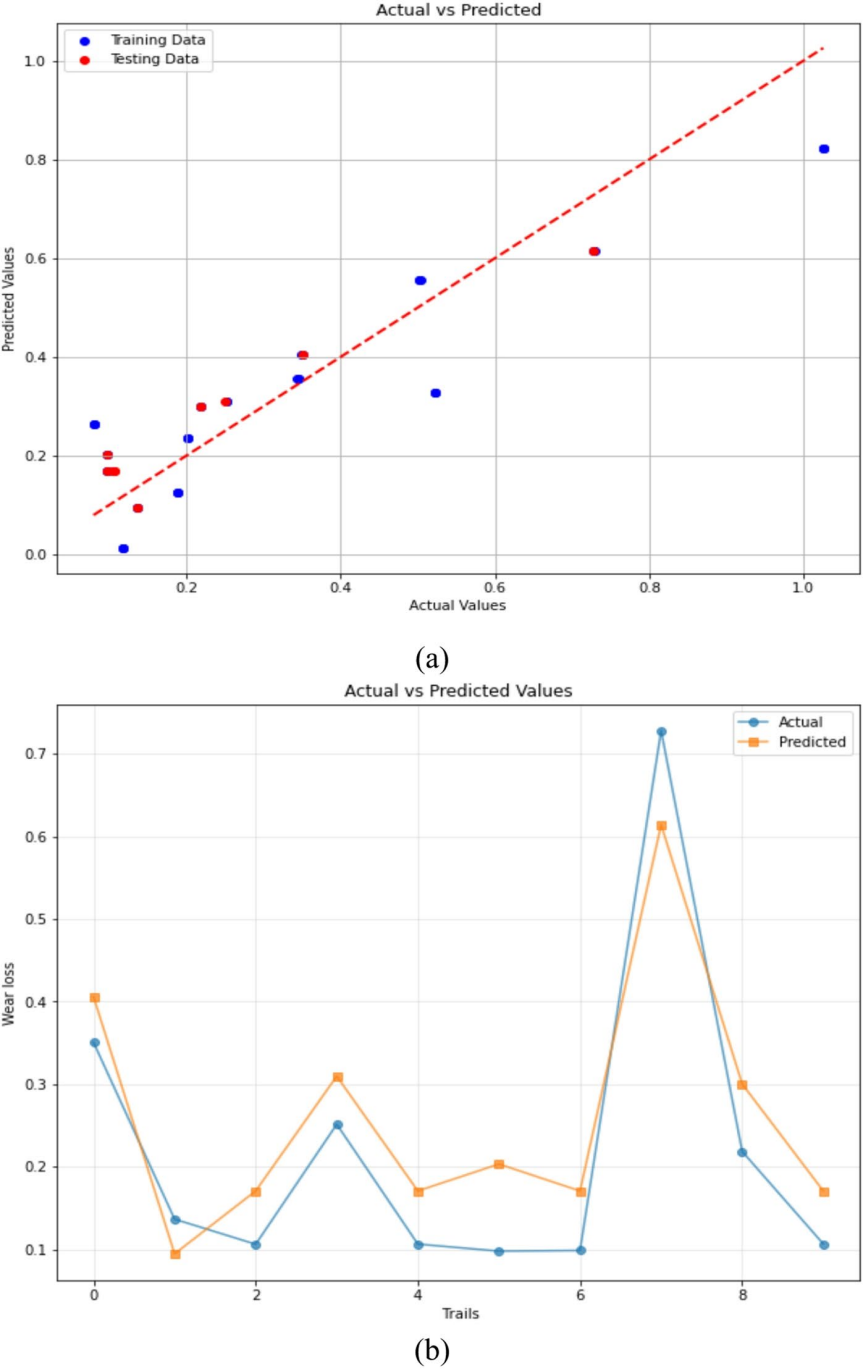
ANN Regression predictions of 3-BAW in µCC-filled H/B F-Ep composites: (**a**) actual vs. predicted wear loss, (**b**) actual and predicted values are compared trial-by-trial.

The Artificial Neural Network regression (ANNR) model’s predictive ability in predicting the 3-BAW behavior of µCC-modified hemp–bamboo/epoxy composites is demonstrated in Fig. 13a and b. The ANNR’s good fitting ability is demonstrated in Fig. 13a, where the predicted wear loss values closely match the actual experimental values. Trial-wise variations are further compared in Fig. 13b, where the ANN shows good generalization with few differences between anticipated and actual wear losses.

The ANN model architecture comprised an input layer (3 neurons) corresponding to µCC content, load, and abrading distance; one hidden layer with 10 neurons using the ReLU activation function; and a single output neuron representing wear loss. Training was performed using the Adam optimizer with a learning rate of 0.001, batch size of 16, and 100 epochs, requiring approximately 3 min for convergence. The model exhibited stable learning behavior with continuous reduction in MSE across epochs, confirming convergence without overfitting. This configuration improves transparency and reproducibility of the ANN setup. Artificial neural networks (ANN) are increasingly being used in tribology. They frequently outperform conventional ML models like LR, decision trees, and SVMs in capturing intricate nonlinear wear-friction correlations and providing precise predictive performance. To properly describe nonlinear input–output interactions, artificial neural networks (ANNs) use a multi-layered design and adaptive learning. Zhu et al.^88^ highlighted the importance of dataset size and showed that ANN predictions of the friction coefficient matched experimental results better than volumetric wear loss for carbon fiber and TiO_2_-reinforced PTFE. Similarly, Ramesh and Suresha^89^ shown that when it comes to estimating the specific wear rate of fiber-reinforced polymer composites, ANN-based models perform better than LR. The current study demonstrated the robustness of the ANN model by predicting 3-BAW in µCC-modified H/B F-Ep composites with a high coefficient of determination (R^2^ = 0.8391) and low error values (RMSE = 0.07523, MAE = 0.07210). Additionally, ANN outperformed traditional models in abrasive wear modeling of rubber composites, achieving up to 96% prediction accuracy^90^. Test size = 0.2, Random state = 42 activation=’relu, optimizer=’adam, learning_rate = 0.00, validation split = 20%, epochs = 100.

#### K-Nearest neighbors (KNN) regression model

**Fig. 14:**
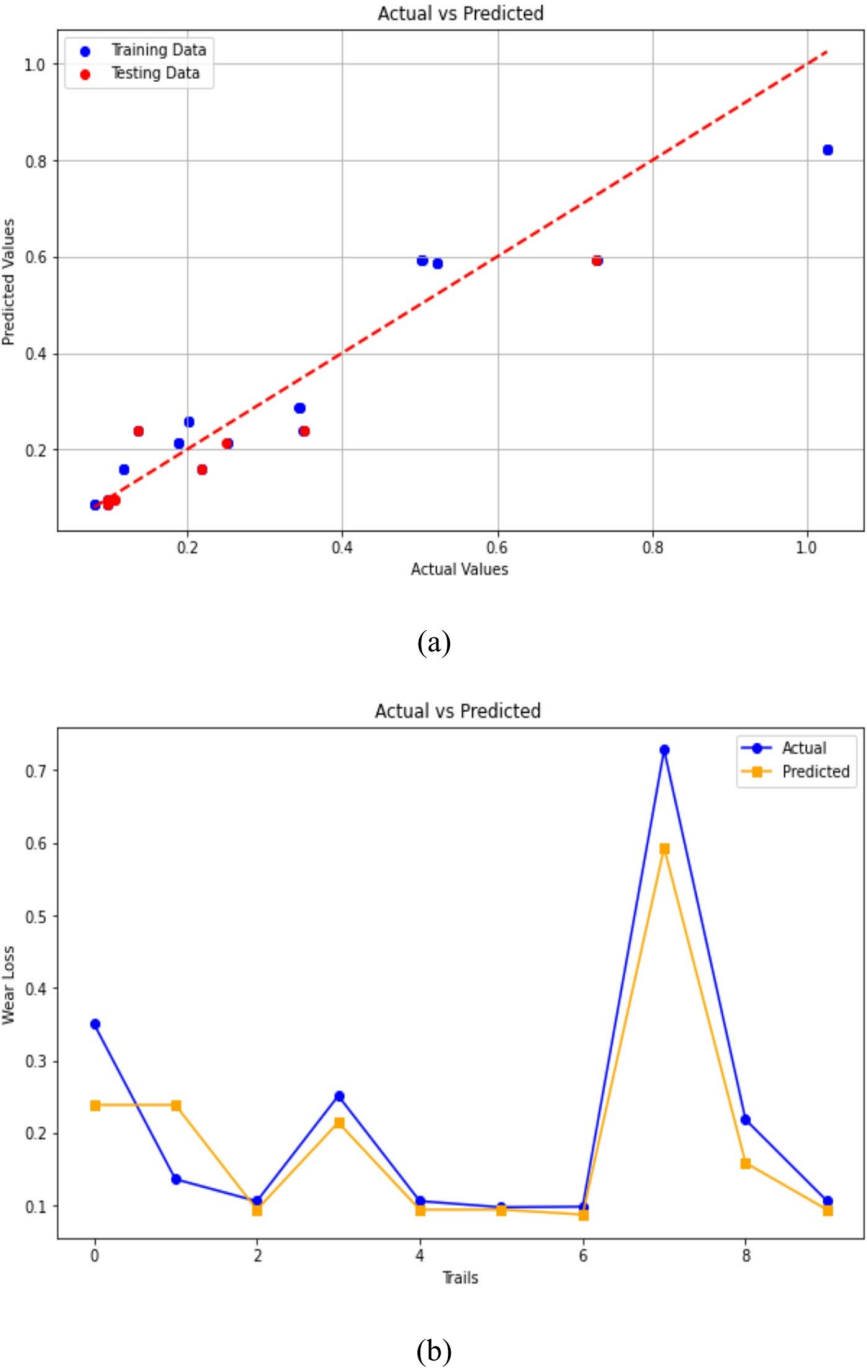
KNN Regression predictions of 3-BAW in µCC-filled H/B F-Ep composites: (**a**) actual vs. predicted wear loss, (**b**) actual and predicted values are compared trial-by-trial.

Figure 14a shows the scatter plot of actual versus predicted wear loss using the K-Nearest Neighbors (KNN) regression model. The model’s predictions and the actual values are reasonably close, as indicated by the fact that most of the points are close to the diagonal line. A few points do, however, diverge, illustrating how KNN relies on adjacent data points to make predictions. The trial-by-trial comparison of actual and predicted values is displayed in Fig. 14b. Despite a few minor variations, the KNN model can track the general wear behavior trend across trials. This illustrates how well KNN learns patterns from local similarities in the dataset. To optimize the model, the KNN underwent k-value tuning using 10-fold cross-validation to determine the best neighborhood size and distance metric. The optimal configuration (k = 5, Euclidean distance) minimized prediction error and enhanced generalization, ensuring that the model effectively captured local nonlinear trends while avoiding overfitting. The test size was set to 0.2, and the random state was fixed at 42.

These findings are consistent with current advances in tribological modeling. According to Machine learning algorithms, specifically XGBoost and Random Forest, have been shown by Sivaraman et al.^91^ to be able to reliably forecast the wear rate (R^2^ = 0.98) and phase formation (98.5% accuracy) of High Entropy Alloy coatings. Their work shows that machine learning (ML) is an efficient method for speeding up coating design and performance enhancement. Test size = 0.2, Random state = 42, No. of Neighbours = 5 and Distance Computation: Euclidean.

The research also emphasizes KNN’s strengths as a distance-based, non-parametric learner that performs very well on datasets with local nonlinear structures, particularly when parameter tweaking (e.g., distance weighting, “k” value) and dataset size are meticulously adjusted^92^. The rise of triboinformatics, which combines tribology and data science to forecast wear and friction using machine learning-driven correlations, was emphasized by Hasan et al.^54^. Their review focuses on how machine learning (ML) facilitates new surface roughness studies and greater understanding of structure–property correlations for advanced materials. Moreover, machine learning techniques such as KNN, RF, SVM, and GBR have demonstrated promise in forecasting intricate wear and friction behavior in larger tribology applications when sufficient data and the appropriate model selection are supplied^93^.

#### Random forest regression

**Fig. 15:**
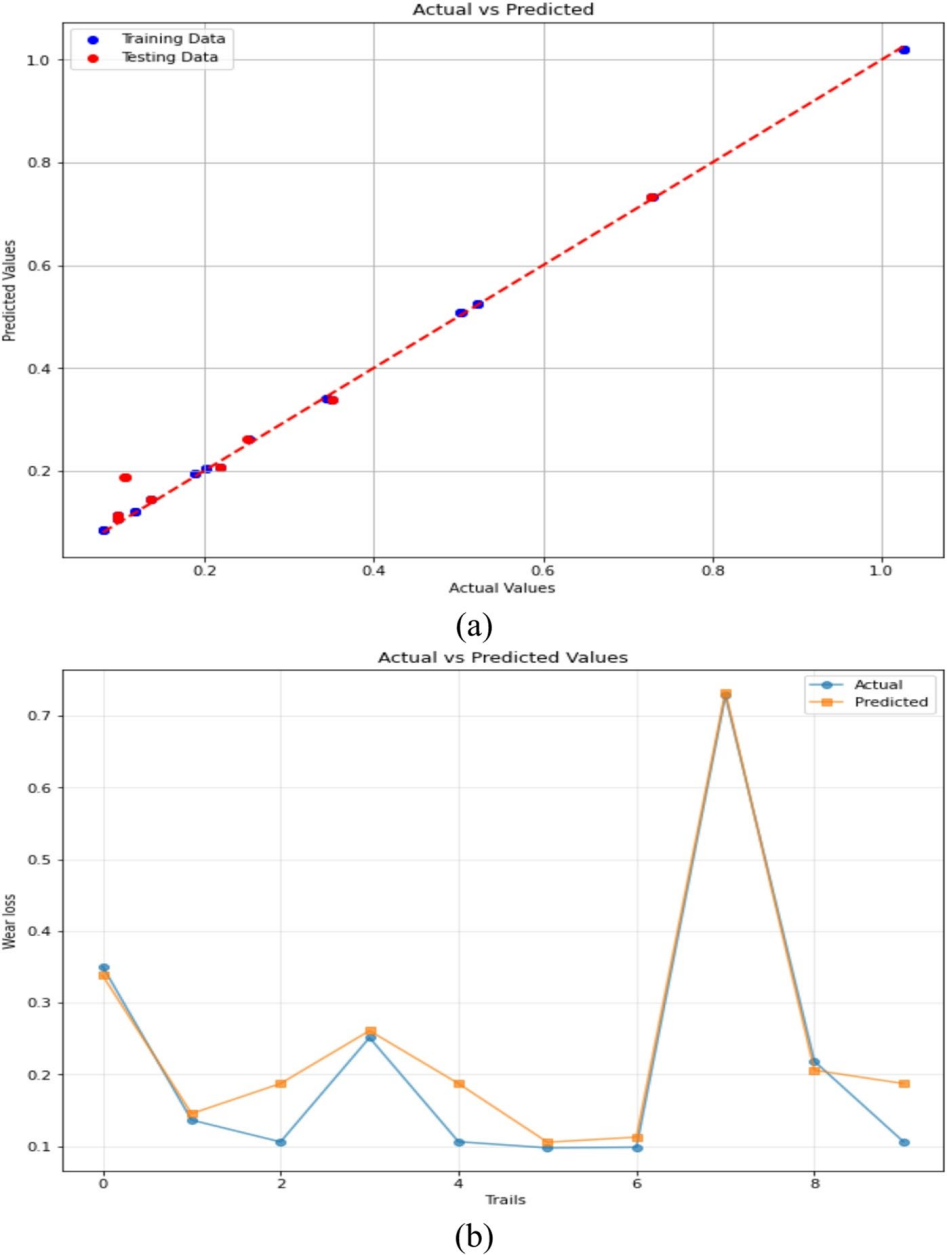
Random Forest Regression predictions of 3-BAW in µCC-filled H/B F-Ep composites: (**a**) actual vs. predicted wear loss, (**b**) actual and predicted values are compared trial-by-trial.

The efficacy of the Random Forest Regression (RFR) model in forecasting the wear loss of 3-BAW in µCC-modified hemp–bamboo/epoxy composites is shown in Fig. 15. The projected values closely follow the diagonal trend line and show a good alignment with the actual experimental values, as illustrated in Fig. 15a. This indicates that the RFR model is resilient and has a high predictive accuracy. This robust association suggests that Random Forest’s ensemble learning strategy, which averages several decision trees, successfully reduces overfitting while capturing the nonlinear relationships between wear loss and the microstructural characteristics of the composite. Test size = 0.2, Random state = 42, max_depth = 10, min_samples_split = 5, and min_samples_leaf = 2.

The model’s dependability is further confirmed by the trial-wise comparison in Fig. 15b, whereby the expected wear values exhibit the same pattern as the actual experimental results, including peak and trough fluctuations. The model accurately depicts both local variations and worldwide trends in wear loss, despite occasional small discrepancies. Because Random Forest can manage complicated variable interactions and noise in experimental datasets, it outperforms simpler models for tribological predictions in natural fiber-based hybrid composites, according to these studies. Recent research in tribology have revealed similar results, with Random Forest achieving great accuracy in forecasting alloy and composite system wear behavior^94–96^. In addition to its predictive accuracy, a feature importance analysis was conducted to interpret the relative influence of µCC content, abrading distance, and applied load on wear behavior. The results, presented alongside the XGBoost model in Fig. 10, indicate that µCC loading contributes most significantly to wear loss reduction, followed by load and abrading distance. This insight not only validates the model’s predictive performance but also provides guidance for optimizing composite formulation and operational parameters for improved tribological performance.

#### Extreme gradient boosting regression

**Fig. 16:**
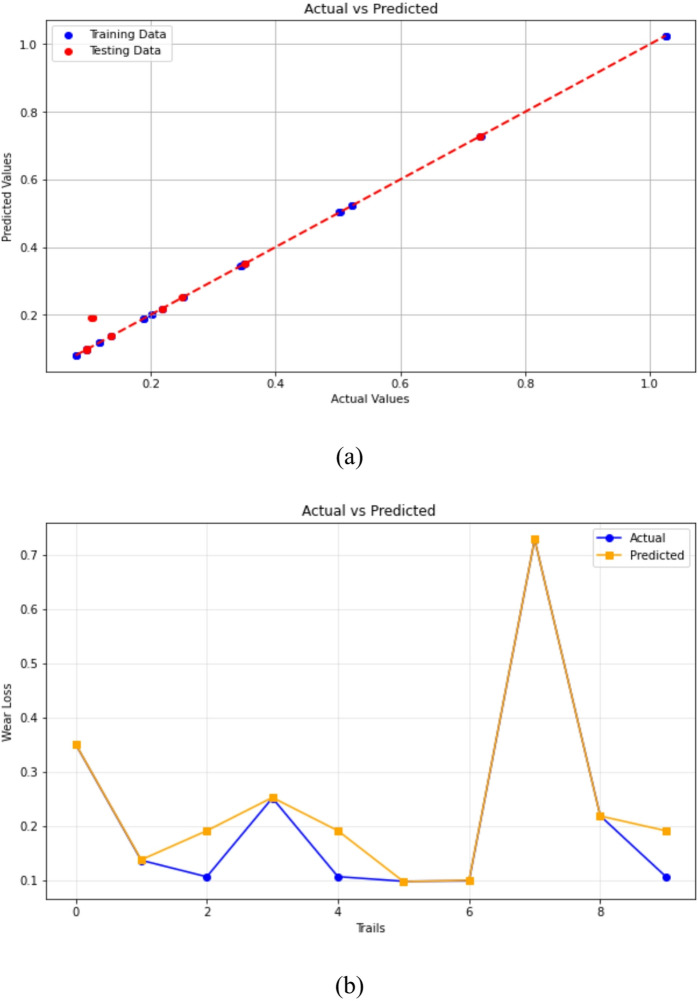
Extreme Gradient Boosting Regression predictions of 3-BAW in µCC-filled H/B F-Ep composites: (**a**) actual vs. predicted wear loss, (**b**) actual and predicted values are compared trial-by-trial.

The Extreme Gradient Boosting (XGBoost) regression model’s capacity to forecast wear loss in 3-BAW µCC-modified hemp–bamboo/epoxy composites are shown in Fig. 16. The predicted values nearly exactly match the actual experimental data along the diagonal line in Fig. 16a, demonstrating XGBoost’s higher accuracy and generalization capabilities over traditional regression methods. This is explained by the gradient boosting framework’s capacity to efficiently capture intricate nonlinear interactions between mechanical and tribological features and fix weak learners’ errors one step at a time. The model was configured with hyperparameters n_estimators = 200, learning_rate = 0.1, max_depth = 4, subsample = 0.8, and colsample_bytree = 0.8, optimized through grid search to balance model complexity and generalization. Although XGBoost achieved the highest predictive accuracy, future studies will integrate SHAP (SHapley Additive exPlanations) analysis to further enhance interpretability by quantifying each feature’s contribution to wear behavior.

The model’s resilience is further demonstrated by the trial-by-trial comparison in Fig. 16b, where XGBoost effectively monitors both local variances and global wear patterns throughout trials. The model’s ability to handle imbalanced datasets and minimize bias–variance trade-offs is demonstrated by the persistent closer agreement it maintains with experimental results, even when there are occasional minor discrepancies at specific peaks. These outcomes are in line with earlier research that indicated XGBoost outperformed alternative ML algorithms in predicting the wear, hardness, and mechanical properties of hybrid alloys and composites^30,97,98^. Remarkably, Xu et al.^99^ found that XGBoost outperformed Random Forest, AdaBoost, and Gradient Boosting in terms of prediction accuracy for the abrasion resistance of manufactured sand concrete. Their study highlights the strong potential of XGBoost in reliably predicting wear-related behavior in construction materials. The efficiency of gradient boosting techniques in tribo-informatics was further emphasized by Hasan et al.^54^, who demonstrated that boosting-based algorithms are more dependable in capturing the extremely nonlinear structure–property–performance correlations in tribology. In bio-epoxy composites, Hiremath et al.^100^ showed that the 0.5 wt% MWCNT loading greatly enhanced wear resistance and surface quality, with Random Forest models obtaining high predictive accuracy (R^2^ = 0.93). Their combined experimental-ML methodology emphasizes how machine learning might improve the prediction of tribological behavior. Thus, the current results demonstrate that XGBoost not only offers the best prediction accuracy of all the tested machine learning models, but it also supports the use of sophisticated ensemble-based techniques for predicting wear loss in natural fiber-reinforced composites, which in turn supports effective material design and optimization. Test size = 0.2, Random state = 42, n_estimators = 200, learning_rate = 0.1, max_depth = 4, subsample = 0.8 and colsample_bytree = 0.8.

The prediction performance of many ML algorithms for determining 3-BAW of µCC-modified H/B F-Ep composites is compiled in Table 6. With an R^2^ of 0.9222 for training and 0.8827 for testing, Linear Regression demonstrated a respectable level of accuracy among the classical models, suggesting that it is a good fit for capturing linear dependencies. The KNN model’s lower R^2^ (0.8674) and marginally better testing MAE (0.0494) reflect its shortcomings with regard to local neighbourhood-based prediction. With comparatively lower R^2^ values and larger errors (MAE = 0.095 training; 0.0721 testing), the ANN model performed moderately, most likely because of under-fitting with a small dataset.

On the other hand, ensemble learning models performed better than the conventional methods. With testing R^2^ = 0.9417 and low RMSE (0.0452), Random Forest provided strong generalization and successfully captured non-linear interactions. With the best training accuracy (R^2^ = 0.9999) and somewhat poor testing performance (R^2^ = 0.6317), gradient boosting may have over fitted. Notably, the XGBoost model outperformed other models in balancing bias and variance, demonstrating exceptional predictive performance with a near-perfect training accuracy (R^2^ = 0.9999) and strong testing accuracy (R^2^ = 0.9387). These results are congruent with those of Xu et al.^99^ and Hiremath et al.^100^, who found that ensemble machine learning models like RF and XGBoost consistently produced better predictions of tribological behavior than either traditional regression or neural models.

**Table 6:** The performance of ML algorithms in predicting abrasive wear loss.

	MAE		MSE		RMSE		*R* ^2^	
	Training	Testing	Training	Testing	Training	Testing	Training	Testing
Linear Regression	0.0614	0.0562	0.00575	0.00422	0.0758	0.0649	0.9222	0.8827
KNN	0.0670	0.0494	0.00729	0.00466	0.085424	0.06827	0.8982	0.8674
ANN	0.0950	0.0721	0.0127	0.00566	0.113	0.07523	0.8216	0.8391
Random Forest	0.00616	0.0313	5.25 × 10^− 5^	0.00204	0.00724	0.0452	0.9992	0.9417
Gradient Boosting	0.00020	0.0626	6.99 × 10^− 8^	0.01295	0.0002	0.1138	0.9999	0.6317
XGB	0.000423	0.0258	2.84 × 10^− 6^	0.002152	0.00053	0.0463	0.9999	0.9387

To ensure reliable predictions and mitigate overfitting, all models were evaluated using 10-fold cross-validation during hyperparameter tuning, and regularization techniques (e.g., controlling tree depth, learning rate, subsampling, and minimum sample constraints) were applied. Testing metrics were calculated on an independent 20% hold-out dataset to provide an unbiased assessment of model performance. Ensemble models, such as Gradient Boosting, Random Forest, and XGBoost, exhibited near-perfect training accuracy (R² ≈ 0.9999), while testing performance was lower, highlighting the potential for overfitting. Key hyperparameters—including max_depth, n_estimators, subsample, colsample_bytree, and minimum samples per leaf—were optimized to balance model complexity and generalization. Error distributions and residual plots were also examined to confirm unbiased predictions across the observed range of wear values.

For Gradient Boosting, the testing R² was 0.6317, indicating limited generalization. Performance can be improved by careful tuning of learning rate, max_depth, and number of estimators, as well as using early stopping to prevent overfitting.

Both Random Forest and XGBoost showed strong testing performance (R² ≈ 0.9417 and 0.9387, respectively), but the discrepancy with near-perfect training values underscores the overfitting risk. Regularization and cross-validation ensured that these models maintained robust generalization. The ANN results showed comparatively higher error, with testing R² (0.8391) slightly exceeding training R² (0.8216). This behavior can arise from limited dataset size and the stochastic nature of ANN training. The network architecture consisted of 3 hidden layers with 16, 12, and 8 neurons, using ReLU activation functions, trained for 500 epochs with a learning rate of 0.01. These hyperparameters influenced predictive accuracy and demonstrate the importance of architecture design and dataset size.

One important strength of the study that deserves greater prominence is its emphasis on sustainable materials. An environmentally conscious approach is demonstrated using bio-based µCC and natural fibers (bamboo/hemp) as fillers, which help to lessen dependency on synthetic fibers and fillers made from petroleum. Significant environmental benefits of these bio-composites include improved resource renewability, reduced carbon footprint, and biodegradability. By emphasizing these features, the manuscript becomes more relevant and in line with worldwide trends toward sustainable materials engineering. Additionally, a clear description of the possible range of applications for these environmentally friendly composites should be provided. They are promising options for wear-intensive engineering applications due to their increased stiffness and wear resistance. In particular, the balance of mechanical performance and environmental sustainability that these composites offer can be very advantageous for automotive components like lightweight body parts, protective panels in construction, and interior panels and trim. There are also promising application areas in other industries that need sustainable yet long-lasting materials, such as consumer goods and athletic gear. By highlighting these opportunities, the manuscript’s impact can be increased by demonstrating the useful, industry-relevant advantages of the created bio-composite system.

## Conclusion and outlook

- This study investigated the effect of microcrystalline cellulose (µCC) incorporation on the three-body abrasive wear (3-BAW) behavior of hemp/bamboo fiber-reinforced epoxy composites, showing that µCC enhanced interfacial bonding (FTIR: intensified hydroxyl and carbonyl peaks), increased hardness, and altered wear mechanisms, with µCC-filled composites exhibiting milder three-body abrasion while unfilled composites suffered severe two-body abrasion.
- Improved wear resistance and reduced wear loss at higher µCC contents are consistent with the Lancaster-Ratner model linking increased hardness and toughness to decreased abrasive wear.
- Advanced machine learning (ML) models predicted 3-BAW behavior effectively, with Extreme Gradient Boosting (XGBoost) and Random Forest capturing nonlinear interactions between µCC content, load, and abrading distance.
- XGBoost achieved R² = 0.9999 (training), 0.9387 (testing), MAE = 0.000423 (training), 0.0258 (testing), RMSE = 0.00053 (training), and 0.0463 (testing), while Random Forest showed training R² = 0.9992 and testing R² = 0.9417, demonstrating reliable prediction and potential to reduce experimental trials.
- Gradient Boosting exhibited lower generalization (testing R² = 0.6317) due to overfitting, and ANN models showed moderate performance, highlighting the influence of dataset size and hyperparameter selection; ML models can predict trends but cannot yet replace mechanistic understanding from experiments.
- The combination of µCC reinforcement and ML-assisted prediction enables accelerated composite design with reduced material and time costs, suitable for applications such as automotive lightweight components, protective panels in construction, interior trims, and other wear-intensive sectors.
- Future work should focus on integrating interpretability techniques (e.g., SHAP analysis) to quantify feature contributions, expanding datasets to improve ANN performance, and exploring real-time, in-situ wear prediction for industrial deployment.

Overall, the synergy of µCC reinforcement and ML modeling provides a sustainable, efficient, and predictive approach for designing high-performance hybrid epoxy composites for wear-critical applications.

## Data Availability

The datasets used and/or analysed during the current study available from the corresponding author on reasonable request.

## References

[1] Mitra, B. C. Environment friendly composite materials: biocomposites and green composites. *Def. Sci. J.* **64**10.14429/dsj.64.7323 (2014).

[2] Uddin, M. H. et al. Nur-E-Alam, advances in natural fiber polymer and PLA composites through artificial intelligence and machine learning integration. *J. Polym. Res.* **32**, 76. 10.1007/s10965-025-04282-7 (2025).

[3] Chandgude, S. & Salunkhe, S. Biofiber-reinforced polymeric hybrid composites: an overview on mechanical and tribological performance. *Polym. Compos.* **41**, 3908–3939. 10.1002/pc.25801 (2020).

[4] Wibowo, C. H., Ariawan, D., Surojo, E. & Sunardi, S. Microcrystalline cellulose as composite reinforcement: assessment and future prospects. *Mater. Sci. Forum Trans. Tech. Publ*. 65–80. 10.4028/p-viYb6d (2024).

[5] Sumesh, K. R., Kanthavel, K. & Kavimani, V. Machinability of hybrid natural fiber reinforced composites with cellulose micro filler incorporation. *J. Compos. Mater.* **54**, 3655–3671. 10.1177/0021998320918020 (2020).

[6] Kabeb, S. M. Environmentally benign High-Performance Composites‐Based hybrid microcrystalline Cellulose/Graphene oxide. *Polym. Adv. Technol.* **35**, e6610. 10.1002/pat.6610 (2024).

[7] Mohammadi, M., Mohamad, H., Rangappa, S. M. & Siengchin, S. Incorporation of nano-fillers on the properties of natural fiber reinforced composites: A review. *Sustain. Mater. Techno*. **14**, e01600. 10.1016/j.susmat.2025.e01600 (2025).

[8] Olanrewaju, O. F., Anaele, J. U. & Kareem, S. A. Experimental and computational approaches to optimizing the development of NFs reinforced polymer composite: A review of optimization strategies. *Sustain. Mater. Techno*. **20**, e01259. 10.1016/j.susmat.2025.e01259 (2025).

[9] Ayana, K. D., Ha, C. S. & Ali, A. Y. Comprehensive overview of wood polymer composite: formulation and technology, properties, interphase modification, and characterization. *Sustain. Mater. Techno*. **40**, e00983. 10.1016/j.susmat.2024.e00983 (2024).

[10] Gates, J. D. Two-body and three-body abrasion: a critical discussion. *Wear* **214**, 139–146. 10.1016/S0043-1648(97)00188-9 (1998).

[11] Stachowiak, G. B. & Stachowiak, G. W. The effects of particle characteristics on three-body abrasive wear. *Wear* **249**, 201–207. 10.1016/S0043-1648(01)00557-9 (2001).

[12] Ohtani, T., Kamasaki, K. & Tanaka, C. On abrasive wear property during three-body abrasion of wood. *Wear* **255**, 60–66. 10.1016/S0043-1648(03)00219-9 (2003).

[13] Sanman, S., Manjunath, A., Prashanth, K. P., Shadakshari, R. & Sunil, S. K. An experimental study on two body abrasive wear behavior of natural fiber reinforced hybrid polymer matrix composites using Taguchi analysis. *Mater. Today Proc.* **72**, 2021–2026. 10.1016/j.matpr.2022.07.400 (2023).

[14] Savaş, S., Gurbanov, N. & Doğan, M. Effect of fiber type, fiber content, and compatibilizer on two-body abrasive wear performance of HDPE matrix composites. *J. Compos. Mater.* **53**, 2743–2760. 10.1177/0021998319839135 (2019).

[15] Kumar, S., Prasad, L., Patel, V. K., Kumain, A. & Yadav, A. Experimental and numerical study on physico-mechanical properties and taguchi’s designed abrasive wear behavior of hemp/nettle‐polyester hybrid composite. *Polym. Compos.* **42**, 6912–6927. 10.1002/pc.26350 (2021).

[16] Suresha, B. & Kumar, K. N. S. Investigations on mechanical and two-body abrasive wear behaviour of glass/carbon fabric reinforced vinyl ester composites. *Mater. Des.* **30**, 2056–2060. 10.1016/j.matdes.2008.08.038 (2009).

[17] Ventura, A. M., Kneissl, L. M., Nunes, S. & Emami, N. Recycled carbon fibers as an alternative reinforcement in UHMWPE composite. Circular economy within polymer tribology. *Sustain. Mater. Techno*. **34**, e00510. 10.1016/j.susmat.2022.e00510 (2022).

[18] Saha, D., Sharma, D. & Bhabani, K. Satapathy. Challenges pertaining to particulate matter emission of toxic formulations and prospects on using green ingredients for sustainable eco-friendly automotive brake composites. *Sustain. Mater. Techno*. **37**, e00680. 10.1016/j.susmat.2023.e00680 (2023).

[19] Kumaresan, K., Chandramohan, G., Senthilkumar, M. & Suresha, B. Dynamic mechanical analysis and three-body wear of carbon–epoxy composite filled with SiC particles. *J. Reinf. Plast. Compos.* **31**, 1435–1448. 10.1177/0731684412459250 (2012).

[20] Manoharan, S., Suresha, B., Bharath, P. B. & Ramadoss, G. Investigations on three-body abrasive wear behaviour of composite brake pad material. *Plast. Polym. Technol.* **3**, 10–18 (2014).

[21] Mishra, V. & Biswas, S. Three-body abrasive wear behavior of short jute fiber reinforced epoxy composites. *Polym. Compos.* **37**, 270–278. 10.1002/pc.23178 (2016).

[22] Rajashekaraiah, H., Mohan, S., Pallathadka, P. K. & Bhimappa, S. Dynamic mechanical analysis and three-body abrasive wear behaviour of thermoplastic copolyester elastomer composites. *Adv. Tribology*. **2014**, 210187. 10.1155/2014/210187 (2014).

[23] Darshan, S. M., Suresha, B. & Jamadar, I. M. Optimization of abrasive wear parameters of Halloysite nanotubes reinforced silk/basalt hybrid epoxy composites using Taguchi approach. *Tribology Ind.* **44**, 253. 10.24874/ti.1131.06.21.08 (2022).

[24] Suresha, B., Hemanth, R. & Udaya Kumar, P. A. Characterization of mechanical and tribological properties of vinyl Ester-Based hybrid green composites. *Hybrid. Fiber Composites: Mater. Manuf. Process. Eng.* 233–263. 10.1002/9783527824571.ch12 (2020).

[25] Kumar, P. A., Suresha, B. & Hemanth, R. Mechanical and tribological behavior of vinyl ester hybrid composites. *Tribology Ind.* **40**10.24874/ti.2018.40.02.12 (2018).

[26] Yuvaraj, G., Ramesh, M. & Rajeshkumar, L. Carbon and cellulose-based nanoparticle-reinforced polymer nanocomposites: a critical review. *Nanomaterials* **13**, 1803. 10.3390/nano13111803 (2023). 37299706 10.3390/nano13111803 PMC10255476

[27] Karuppusamy, M. et al. A review of machine learning applications in polymer composites: advancements, challenges, and future prospects. *J. Mater. Chem. Mater.*10.1039/d5ta00982k (2025).

[28] Sathiyamurthy, S., Saravanakumar, S. & Vinoth, V. Enhancing tribological performance of hybrid fiber-reinforced composites through machine learning and response surface methodology. *J. Reinf. Plast. Compos.* 07316844241256421. 10.1177/07316844241256421 (2024).

[29] Satish Kumar, D. & Rajmohan, M. Optimizing wear behavior of epoxy composites using response surface methodology and artificial neural networks. *Polym. Compos.* **40**, 2812–2818. 10.1002/pc.25089 (2019).

[30] Mohammed, A. J., Mohammed, A. S. & Mohammed, A. S. Prediction of tribological properties of UHMWPE/SiC polymer composites using machine learning techniques. *Polym. (Basel)*. **15**, 4057. 10.3390/polym15204057 (2023). 10.3390/polym15204057 PMC10610110 37896301

[31] Malashin, I., Tynchenko, V., Gantimurov, A., Nelyub, V. & Borodulin, A. Boosting-Based machine learning applications in polymer science: A review. *Polym. (Basel)*. **17**, 499. 10.3390/polym17040499 (2025). 10.3390/polym17040499 PMC11859903 40006161

[32] Ibrahim, M. A. et al. Hybrid artificial intelligence models with multi objective optimization for prediction of tribological behavior of polytetrafluoroethylene matrix composites. *Appl. Sci.* **12**, 8671. 10.3390/app12178671 (2022).

[33] Prabhu, R., Mendonca, S., Bellairu, P. K., D’Souza, R. & Bhat, T. Modeling and analysis of TiO2 filler’s impact on specific wear rate in flax fiber-reinforced epoxy composite under abrasive wear using Taguchi approach. *Multidiscipline Model. Mater. Struct.* **20**, 546–557. 10.1108/MMMS-10-2023-0342 (2024).

[34] Şahin, Y. & Şahin, F. Effects of process factors on tribological behaviour of epoxy composites including Al2O3 nano particles: a comparative study on multi-regression analysis and artificial neural network. *Adv. Mater. Process. Technol.* **8**, 2007–2021. 10.1080/2374068X.2021.1878712 (2022).

[35] Singh, M., Dodla, S., Gautam, R. K. & Chauhan, V. Enhancement of mechanical and tribological properties in glass fiber-reinforced polymer composites with multi-walled carbon nanotubes and ANN-based COF prediction. *Compos. Interfaces*. **32**, 439–459. 10.1080/09276440.2024.2417164 (2025).

[36] Jain, P., Joshi, U., Joshi, A., Patel, V. & Thakor, S. Comparative analysis of machine learning techniques for predicting wear and friction properties of MWCNT reinforced PMMA nanocomposites. *Ain Shams Eng. J.* **15**, 102895. 10.1016/j.asej.2024.102895 (2024).

[37] Pan, Y. et al. Characterization of epoxy composites reinforced with wax encapsulated microcrystalline cellulose. Polymers, 8 (12), (2016) 415. (2016). 10.3390/polym8120415 10.3390/polym8120415 PMC6432487 30974692

[38] Szatkowski, P., Szatkowska, M., Gralewski, J., Czechowski, L. & Kedziora, S. Biocomposites with epoxy resin matrix modified with ingredients of natural origin. *Materials* **15** (20), 7167. 10.3390/ma15207167 (2022). 36295235 10.3390/ma15207167 PMC9611067

[39] Mittal, V., Saini, R. & Sinha, S. Natural fiber-mediated epoxy composites–a review. *Compos. Part. B: Eng.* **99**, 425–435. 10.1016/j.compositesb.2016.06.051 (2016).

[40] Stanley, W. F., Bandaru, A. K., Rana, S., Parveen, S. & Pichandi, S. Mechanical, dynamic-mechanical and wear performance of novel non-crimp glass fabric-reinforced liquid thermoplastic composites filled with cellulose microcrystals. *Mater. Design*. **212**, 110276. 10.1016/j.matdes.2021.110276 (2021).

[41] Giridharan, K. et al. Investigation of dry sliding wear and mechanical properties of hybrid epoxy composites reinforced with pineapple leaf and roselle fibers. *Sci. Rep.* **15** (1), 27991. 10.1038/s41598-025-10431-1 (2025). 40744955 10.1038/s41598-025-10431-1 PMC12314131

[42] Yusuf, J., Sapuan, S. M., Rashid, U., Ilyas, R. A. & Hassan, M. R. Thermal, mechanical, and morphological properties of oil palm cellulose nanofibril reinforced green epoxy nanocomposites. *Int. J. Biol. Macromol.* **278**, 134421. 10.1016/j.ijbiomac.2024.134421 (2024). 39227276 10.1016/j.ijbiomac.2024.134421

[43] Mahmud, M. Z. A., Rabbi, S. F., Islam, M. D. & Hossain, N. Synthesis and applications of natural fiber-reinforced epoxy composites: A comprehensive review. *SPE Polym.* **6** (1), e10161. 10.1002/pls2.10161 (2025).

[44] ASTM. *Standard Test Method for Rubber property-durometer Hardness* 2240–15e1 (ASTM International, 2015).

[45] Test Method for Measuring Abrasion Using the Dry Sand/Rubber Wheel Apparatus. (2021). 10.1520/G0065-16R21

[46] Montgomery, D. C., Peck, E. A. & Vining, G. G. *Introduction To Linear Regression Analysis* 6th edn (Wiley, 2021).

[47] Liang, Y., Hu, S., Guo, W. & Tang, H. Abrasive tool wear prediction based on an improved hybrid difference grey Wolf algorithm for optimizing SVM. *Measurement* **187**, 110247 (2022).

[48] Bermejo, S. & Cabestany, J. Adaptive soft k-nearest-neighbour classifiers. *Pattern Recognit.* **33**, 1999–2005. 10.1016/S0031-3203(99)00186-7 (2000).

[49] Srisuradetchai, P. & Suksrikran, K. Random kernel k-nearest neighbors regression. *Front. Big Data*. **7**, 1402384 (2024). 39011467 10.3389/fdata.2024.1402384 PMC11246867

[50] Zhang, L., Liu, Q., Yang, W., Wei, N. & Dong, D. An improved k-nearest neighbor model for short-term traffic flow prediction. *Procedia-Social Behav. Sci.* **96**, 653–662. 10.1016/j.sbspro.2013.08.076 (2013).

[51] Miguez, R., Georgiopoulos, M. & Kaylani, A. A genetically engineered probabilistic neural network. *Nonlinear Anal. Theory Methods Appl.* **73**, 1783–1791. 10.1016/j.na.2010.04.080 (2010).

[52] Liu, X., Tian, S., Tao, F. & Yu, W. A review of artificial neural networks in the constitutive modeling of composite materials. *Compos. B Eng.* **224**, 109152. 10.1016/j.compositesb.2021.109152 (2021).

[53] Kolev, M. A machine learning software in python for predicting the friction coefficient of porous Al-based composites with extreme gradient boosting. *Softw. Impacts*. **17**, 100531. 10.1016/j.simpa.2023.100531 (2023).

[54] Hasan, M. S. & Nosonovsky, M. Machine learning algorithms and data topology methods for tribology. *Surf. Innov.* **10**, 229–242. 10.1680/jsuin.22.00027 (2022).

[55] Wang, Q., Wang, X., Zhang, X., Li, S. & Wang, T. Tribological properties study and prediction of PTFE composites based on experiments and machine learning. *Tribol Int.* **188**, 108815. 10.1016/j.triboint.2023.108815 (2023).

[56] James, G., Witten, D., Hastie, T. & Tibshirani, R. *An Introduction To Statistical Learning: with Applications in R* Vol. 103 (springer, 2013).

[57] Friedman, J. H. Greedy function approximation: A gradient boosting machine. *Annals Stat.* **29**10.1214/aos/1013203451 (2001).

[58] He, X. et al. in: Practical Lessons from Predicting Clicks on Ads at Facebook, in, ACM Press, New York, New York, USA, pp. 1–9, (2014). 10.1145/2648584.2648589

[59] Tavadi, A. R. et al. Experimental analysis of mechanical properties and FTIR analysis of Areca fiber-reinforced epoxy composites incorporating Al2O3. *J. Ind. Text.* **55**, 15280837241313217. 10.1177/15280837241313217 (2025).

[60] Gopal, R., Aananthakumar, K., Nellaiappan, T. A., Padmesh, R. & Arun, E. Development of coir, glass fiber and sic-reinforced epoxy resin hybrid composites for Building panels applications. *Cellul. Chem. Technol.* **58** (9–10), 1003–1013 (2014).

[61] Zhang, K. et al. Thermal and mechanical properties of bamboo fiber reinforced epoxy composites. *Polymers* **10** (6), 608. 10.3390/polym10060608 (2018). 30966642 10.3390/polym10060608 PMC6404121

[62] Luo, G. et al. Dynamic adhesion of polyamide 6, 6 cord/rubber composites in H-pull tests: fatigue evolution, life prediction and methodology evaluation. *Polym. Test.* **111**, 107586. https://doi.org/10.1016/j. polymer testing.2022.107586 (2022).

[63] Poopakdee, N. & Thammawichai, W. The effects of the crystallinity index of cellulose on the flexural properties of hybrid-cellulose epoxy composites. *J. Met. Mater. Minerals*. **34** (3), 1902–1902. 10.55713/jmmm.v34i3.1902 (2024).

[64] Lou, C. et al. Self-extracted corn-stalk cellulose/epoxy resin composites. *Sci. Rep.* **12**, 20968. 10.1038/s41598-022-25695-0 (2022). 36471157 10.1038/s41598-022-25695-0 PMC9722901

[65] Panchal, M. et al. Spectroscopic analysis of natural fiber/epoxy composites, in: Handbook of Epoxy/Fiber Composites, Springer, : 1–36. 10.1007/978-981-19-3603-6_21. (2022).

[66] Bandaru, A. K., Pichandi, S., Ma, H., Panchal, M. & Gujjala, R. Effect of microcrystalline cellulose on the mechanical properties of flax reinforced methylmethacrylate and urethane acrylate composites. *J. Mater. Sci.* **59**, 2872–2892. 10.1007/s10853-024-09349-2 (2024).

[67] Lupidi, G., Pastore, G., Marcantoni, E. & Gabrielli, S. Recent developments in chemical derivatization of microcrystalline cellulose: Pre-treatments, functionalization, and applications, Molecules 28 2009. (2023). 10.3390/molecules28052009 10.3390/molecules28052009 PMC10004355 36903254

[68] Rathnayake, W. S. M., Karunanayake, L., Samarasekara, A. & Amarasinghe, D. A. S. Fabrication and characterization of polypropylene microcrystalline cellulose based composites with enhanced compatibility, in: 2019 Moratuwa Engineering Research Conference (MERCon), IEEE, : pp. 354–359. (2019). 10.1109/MERCon.2019.8818914

[69] Chowdhury, S. G. et al. Morphology and physico-mechanical threshold of α-cellulose as filler in an E-SBR composite. *Molecules* **26** (3), 694. 10.3390/molecules26030694 (2021). 33525731 10.3390/molecules26030694 PMC7866042

[70] Dastjerdi, S., Naeijian, F., Akgöz, B. & Civalek, Ö. On the mechanical analysis of microcrystalline cellulose sheets. *Int. J. Eng. Sci.* **166**, 103500. 10.1016/j.ijengsci.2021.103500 (2021).

[71] Yano, S., Hsu, Y. I. & Uyama, H. Improvement of mechanical and thermal properties of epoxy composites with citric acid modified and defibrated cellulose filler. *Polym. Degrad. Stab.* **240**, 111482. 10.1016/j.polymdegradstab.2025.111482 (2025).

[72] Shalwan, A., Alajmi, F. M. & Alajmi, N. The impact of filler content on mechanical and micro-structural characterization of graphite-epoxy composites. *J. Mater. Sci. Chem. Eng.* **10** (6), 19–29. 10.4236/msce.2022.106003 (2022).

[73] Friedrich, K., Zhang, Z. & Klein, P. Wear of polymer composites. *Wear–Materials Mech. Pract.* 269–290. 10.1002/9780470017029.ch11 (2005).

[74] Prasad, A. V. R. & Rao, K. M. Mechanical properties of natural fibre reinforced polyester composites: Jowar, Sisal and bamboo. *Mater. Des.* **32**, 4658–4663. 10.1016/j.matdes.2011.03.015 (2011).

[75] Bijwe, J., Indumathi, J. & Ghosh, A. K. On the abrasive wear behaviour of fabric-reinforced polyetherimide composites. *Wear* **253**, 768–777. 10.1016/S0043-1648(02)00169-2 (2002).

[76] Suresha, B., Seetharamu, S. & Kumaran, P. S. Investigations on the influence of graphite filler on dry sliding wear and abrasive wear behaviour of carbon fabric reinforced epoxy composites. *Wear* **267**, 1405–1414. 10.1016/j.wear.2009.01.026 (2009).

[77] Baydoun, S., Fouvry, S. & Descartes, S. Modeling contact size effect on fretting wear: a combined contact oxygenation-third body approach. *Wear* **488**, 204168. 10.1016/j.wear.2021.204168 (2022).

[78] Mohapatra, D. K., Deo, C. R., Mishra, P. & Mishra, C. Influence of load and sliding velocity on abrasive wear of polyester composites reinforced with bio-particulates as filler material. *Trans. Indian Inst. Met.* **77** (8), 2053–2062. 10.1007/s12666-024-03283-2 (2024).

[79] Archard, J. Contact and rubbing of flat surfaces. *J. Appl. Phys.* **24**, 981–988. 10.1063/1.1721448 (1953).

[80] Sharma, S., Bijwe, J., Panier, S. & Sharma, M. Abrasive wear performance of SiC-UHMWPE nano-composites–Influence of amount and size. *Wear* **332**, 863–871. 10.1016/j.wear.2015.01.012 (2015).

[81] Suresha, B., Vidyashree, S. & Bettegowda, H. Effect of filler materials on abrasive wear performance of Glass/Epoxy composites. *Tribology Ind.* **44**, 111. 10.24874/ti.1386.10.22.01 (2023).

[82] Suresha, B., Rajamurugan, G. & Megalingam, A. Mechanical and abrasive wear behavior of cenosphere filled carbon reinforced epoxy composites using Taguchi-Grey relational analysis. *Mater. Res. Express*. **6**, 015307. 10.1088/2053-1591/aae5dc (2018).

[83] Yan, H. et al. Machine Learning-Based prediction of tribological properties of epoxy composite coating. *Polym. (Basel)*. **17**, 282. 10.3390/polym17030282 (2025). 10.3390/polym17030282 PMC11820021 39940485

[84] Yan, Y., Du, J., Ren, S. & Shao, M. Prediction of the tribological properties of polytetrafluoroethylene composites based on experiments and machine learning. *Polym. (Basel)*. **16**, 356. 10.3390/polym16030356 (2024). 10.3390/polym16030356 PMC10857071 38337245

[85] Fathi, R., Chen, M., Abdallah, M. & Saleh, B. Wear prediction of functionally graded composites using machine learning. *Materials* **17**, 4523. 10.3390/ma17184523 (2024). 39336265 10.3390/ma17184523 PMC11433472

[86] Singh, K. S. K., Kumar, S. & Singh, K. K. Computational data-driven based optimization of tribological performance of graphene filled glass fiber reinforced polymer composite using machine learning approach. *Mater. Today Proc.* **66**, 3838–3846. 10.1016/j.matpr.2022.06.253 (2022).

[87] Marian, M. & Tremmel, S. Current trends and applications of machine learning in tribology—A review. *Lubricants* **9**, 86. 10.3390/lubricants9090086 (2021).

[88] Zhu, J., Shi, Y., Feng, X., Wang, H. & Lu, X. Prediction on tribological properties of carbon fiber and TiO2 synergistic reinforced polytetrafluoroethylene composites with artificial neural networks. *Mater. Des.* **30**, 1042–1049. 10.1016/j.matdes.2008.06.045 (2009).

[89] Ramesh, B. N. & Suresha, B. Optimization of tribological parameters in abrasive wear mode of carbon-epoxy hybrid composites. *Mater. Des.* **59**, 38–49. 10.1016/j.matdes.2014.02.023 (2014).

[90] Arul Marcel Moshi, A., Sundara Bharathi, S. R. & Manikandan, K. R. Effective utilization of optimization algorithms on machining operations. *Indian J. Eng. Mater. Sci. (IJEMS)*. **29**, 155–168 (2022).

[91] Sivaraman, S., Radhika, N. & Khan, M. A. Machine Learning-Driven prediction of wear rate and phase formation in high entropy alloy coatings for enhanced durability and performance. *IEEE Access.*10.1109/ACCESS.2025.3542507 (2025).

[92] Seyghaly, R., Garcia, J., Masip-Bruin, X. & Kuljanin, J. SBNNR: small-size bat-optimized KNN regression. *Future Internet*. **16**, 422. 10.3390/fi16110422 (2024).

[93] Paturi, U. M. R., Palakurthy, S. T. & Reddy, N. S. The role of machine learning in tribology: A systematic review. *Arch. Comput. Methods Eng.* **30**, 1345–1397. 10.1007/s11831-022-09841-5 (2023).

[94] Hao, Z. H., Feng, P., Zhang, S. & Zhai, Y. Machine learning for predicting fiber-reinforced polymer durability: A critical review and future directions. *Compos. B Eng.* **303**, 112587. 10.1016/j.compositesb.2025.112587 (2025).

[95] Arumugam, S. et al. Machine Learning-based investigation of wear and frictional behavior in Graphite-reinforced aluminum nanocomposites. *NanoWorld J.* **9**, S278–S287. 10.17756/nwj.2023-s3-054 (2023).

[96] Jatavallabhula, J. K., Shabana, S. & Pappula, B. Development and evaluation of machine learning based predictive models for tribological properties of blended coatings at elevated temperature. *J. Bio-and Tribo-Corrosion*. **11**, 25. 10.1007/s40735-025-00952-7 (2025).

[97] Katiyar, J. K. & Mohammed, A. S. Physical, tribological and mechanical properties of polymer composite coating on silicon wafer. *Tribol Int.* **165**, 107307. 10.1016/j.triboint.2021.107307 (2022).

[98] Çevik, Z. A. & Sarıışık, G. Learning-Driven optimization of machining parameters for Nanoclay-Glass fiber Polymer-Reinforced composites for performance prediction and process enhancement. *J. Tribol*. **147**, 091117. 10.1115/1.4068354 (2025).

[99] Xu, X. et al. Experimental study and machine Learning-Based prediction of the abrasion resistance of manufactured sand concrete. *Buildings* **14**10.3390/buildings14113433 (2024).

[100] Hiremath, P. et al. Comprehensive experimental optimization and Image-Driven machine learning prediction of tribological performance in MWCNT-Reinforced Bio-Based epoxy nanocomposites. *J. Compos. Sci.* **9**10.3390/jcs9080385 (2025).

